# Covering complete proteomes with X-ray structures: a current snapshot

**DOI:** 10.1107/S1399004714019427

**Published:** 2014-10-23

**Authors:** Marcin J. Mizianty, Xiao Fan, Jing Yan, Eric Chalmers, Christopher Woloschuk, Andrzej Joachimiak, Lukasz Kurgan

**Affiliations:** a Electrical and Computer Engineering, University of Alberta, Edmonton, Alberta T6G 2V4, Canada; b Midwest Center for Structural Genomics, Argonne National Laboratory, Argonne, IL 60439, USA

**Keywords:** crystallization propensity, proteome coverage, *fDETECT*

## Abstract

Structural genomics programs have developed and applied structure-determination pipelines to a wide range of protein targets, facilitating the visualization of macromolecular interactions and the understanding of their molecular and biochemical functions. The fundamental question of whether three-dimensional structures of all proteins and all functional annotations can be determined using X-ray crystallography is investigated. A first-of-its-kind large-scale analysis of crystallization propensity for all proteins encoded in 1953 fully sequenced genomes was performed. It is shown that current X-ray crystallographic knowhow combined with homology modeling can provide structures for 25% of modeling families (protein clusters for which structural models can be obtained through homology modeling), with at least one structural model produced for each Gene Ontology functional annotation. The coverage varies between superkingdoms, with 19% for eukaryotes, 35% for bacteria and 49% for archaea, and with those of viruses following the coverage values of their hosts. It is shown that the crystallization propensities of proteomes from the taxonomic superkingdoms are distinct. The use of knowledge-based target selection is shown to substantially increase the ability to produce X-ray structures. It is demonstrated that the human proteome has one of the highest attainable coverage values among eukaryotes, and GPCR membrane proteins suitable for X-ray structure determination were determined.

## Abstract 2

The current and the attainable coverage by X-ray structures of proteins and their functions on the scale of the ‘protein universe’ are estimated. A detailed analysis of the coverage across nearly 2000 proteomes from all superkingdoms of life and functional annotations is performed, with particular focus on the human proteome and the family of GPCR proteins.

## 1. Introduction   

Knowledge of the three-dimensional structures of proteins is essential for understanding biological processes (Harrison, 2004 ▶; Chang *et al.*, 2013 ▶). Structures help to explain molecular and biochemical functions, visualize details of macromolecular interactions, facilitate understanding of underlying biochemical mechanisms and define biological concepts. Structural biology has demonstrated remarkable progress over the past two decades (Joachimiak, 2009 ▶; Berman *et al.*, 2012 ▶). The human genome and follow-up sequencing projects have revolutionized biology and medicine (Ball *et al.*, 2012 ▶; Wilson, 2012 ▶; Edwards *et al.*, 2013 ▶). Structural genomics (SG) programs take advantage of this genomic information that is rapidly becoming available for an increasingly larger set of organisms to evaluate the feasibility of determining the structures of a majority of protein families. The challenge is formidable, as the number of protein sequences continues to increase exponentially (Levitt, 2007 ▶, 2009 ▶; Kolodny *et al.*, 2013 ▶). The SG programs have developed and applied structure-determination pipelines to a wide range of protein targets, including membrane proteins (Pieper *et al.*, 2013 ▶), and have used successes and failures to evaluate the feasibility of structure determination (Structural Genomics Consortium *et al.*, 2008 ▶). With over a decade of efforts which have resulted in the accumulation of high-quality data (Berman *et al.*, 2009 ▶; Gabanyi *et al.*, 2011 ▶), the question arises as to how feasible it is to obtain structural models for all protein families found in living organisms.

According to the Protein Data Bank (PDB; Berman *et al.*, 2000 ▶), nearly 90% of protein structures were determined by X-ray crystallography, with further contributions from NMR and electron microscopy (EM). This motivates our focus on the dominant crystallization-based structure determination. Large-scale studies of the crystallization propensities of proteins were initiated with the advent of the comprehensive and well annotated experiments carried out by SG centers (Christendat *et al.*, 2000 ▶; Lesley *et al.*, 2002 ▶), and the first open database resources accessible to the scientific community were created in the early 2000s (Rodrigues & Hubbard, 2003 ▶). These resources were combined into centralized databases, including TargetDB (Chen *et al.*, 2004 ▶) and PepcDB (Protein Expression Purification and Crystallization DataBase; Kouranov *et al.*, 2006 ▶), which were recently combined in the TargetTrack knowledgebase (Berman *et al.*, 2009 ▶; Gabanyi *et al.*, 2011 ▶). Initial efforts to analyze these data concentrated on the characteristics of proteins which could be linked to crystallization successes and failures (Christendat *et al.*, 2000 ▶; Rodrigues & Hubbard, 2003 ▶; Goh *et al.*, 2004 ▶; Canaves *et al.*, 2004 ▶; Kantardjieff & Rupp, 2004 ▶; Kantardjieff *et al.*, 2004 ▶; Oldfield *et al.*, 2005 ▶; Chandonia *et al.*, 2006 ▶). These efforts were soon followed by the development of methods that predict the crystallization outcome of proteins: *SECRET* (Smialowski *et al.*, 2006 ▶), *OB-Score* (Overton & Barton, 2006 ▶), *CRYSTALP* (Chen *et al.*, 2007 ▶), *XtalPred* (Slabinski, Jaroszewski, Rodrigues *et al.*, 2007 ▶; Slabinski, Jaroszewski, Rychlewski *et al.*, 2007 ▶), *ParCrys* (Overton *et al.*, 2008 ▶), *CRYSTALP*2 (Kurgan *et al.*, 2009 ▶), *MetaPPCP* (Mizianty & Kurgan, 2009 ▶), *PXS* (Price *et al.*, 2009 ▶), *SVMCrys* (Kandaswamy *et al.*, 2010 ▶), the MCSG Z-score (Babnigg & Joachimiak, 2010 ▶), *PPCPred* (Mizianty & Kurgan, 2011 ▶), *XANNpred* (Overton *et al.*, 2011 ▶), *CRYSPred* (Mizianty & Kurgan, 2012 ▶), *SCMCRYS* (Charoen­kwan *et al.*, 2013 ▶) and *XtalPred-RF* (Jahandideh *et al.*, 2014 ▶). Recent results reveal that the predictive performance of these methods deteriorates over time as new data are being added and new crystallization and structure-determination protocols are being developed and implemented (Mizianty & Kurgan, 2011 ▶). This calls for intermittent development and advancement of new and existing crystallization propensity predictors. Additionally, new methods must be computationally efficient to handle the exponentially growing protein-sequence space. To this end, we developed an accurate and highly efficient method for the fast determination of the eligibility of targets for crystallization (*fDETECT*) and utilized it to investigate crystallization propensity and the resulting coverage by X-ray structures of the current snapshot of the protein universe.

Protein structure can be also predicted computationally and such efforts have recently been executed on a genome scale (Xu & Zhang, 2013 ▶). Arguably, the most promising computational approaches are based on homology modeling (Ginalski, 2006 ▶). These methods rely on previously solved structures of similar proteins, so-called templates, and the assumption that two proteins that have similar sequences also have similar structures. They work well since despite the large number of unique protein sequences, there is a finite and much lower number of structural motifs/domains that these sequences fold into (Wolf *et al.*, 2000 ▶; Vitkup *et al.*, 2001 ▶; Koonin *et al.*, 2002 ▶; Liu & Rost, 2002 ▶). Several studies have demonstrated that homology-modeling methods predict the structure accurately if the sequence identity between a query protein and a template is at least 30% (Baker & Sali, 2001 ▶; Nair *et al.*, 2009 ▶; Gront *et al.*, 2012 ▶) and have a modest chance of success for sequence identities above 25% (Ginalski, 2006 ▶; Gront *et al.*, 2012 ▶).

We performed a first-of-its-kind large-scale analysis covering all (nearly 2000) known complete proteomes (the sets of proteins thought to be expressed by an organism whose genome has been completely sequenced, as defined in UniProt; UniProt Consortium, 2012 ▶) and all functional and localization annotations available in Gene Ontology (GO; Ashburner *et al.*, 2000 ▶) for the corresponding proteins. This analysis provides interesting insights into our ability to obtain X-ray structures across various proteomes and superkingdoms of life, and quantifies the contributions of X-ray crystallo­graphy and homology modeling towards the ultimate goal of solving the protein-structure space.

## 2. Materials and methods   

### 2.1. Data sets   

We used the training and test benchmark data sets introduced in previous work (Mizianty & Kurgan, 2011 ▶) to design a new crystallization propensity predictor. To allow the accurate prediction of native protein sequences (collected from UniProt) we removed affinity tags (Waugh, 2005 ▶) from these data sets. The sequence identity between chains categorized with the same prediction outcome (crystallizable *versus* noncrystallizable) in the training and test sets is below 25%; this is in line with the evaluation protocols performed in prior related studies (Mizianty & Kurgan, 2011 ▶; Overton *et al.*, 2008 ▶). To further evaluate our predictor, we used protein structures solved by X-ray crystallography that were deposited in the PDB (Berman *et al.*, 2000 ▶) between 1 January 1993 and 31 December 2012 which have resolution no lower than 3.5 Å. We filtered out redundant chains, leaving the chain from the structure with the highest resolution. The corresponding PDB data set consists of 50 138 nonredundant chains from 44 671 X-ray structures. The UniProt data set consists of 9 586 243 proteins (8 652 940 nonredundant) from 1953 complete proteomes (106 archaea, 1043 bacteria, 265 eukaryotes and 539 viruses) collected from release 2012_07 of UniProt. The proteomes were assigned to their taxonomic lineage based on the NCBI BioSystems database (Geer *et al.*, 2010 ▶). The considered data sets are summarized in Supplementary Table S1^1^. We also annotated proteins with Gene Ontology (Ashburner *et al.*, 2000 ▶) annotations where available.

### 2.2. Coverage by the X-ray structures and homology modeling   

To incorporate homology modeling into our analysis of the attainable coverage by the X-ray structures, we clustered the UniProt data set using the *UClust* algorithm (Edgar, 2010 ▶). *UClust* could not process sequences longer than 10 000 amino acids and thus they were removed from the analysis. We utilized several thresholds of protein identity, including 50%, which we used to group functionally similar chains (Addou *et al.*, 2009 ▶; Rentzsch & Orengo, 2013 ▶), 30%, which allows accurate homology modeling (Baker & Sali, 2001 ▶; Nair *et al.*, 2009 ▶; Gront *et al.*, 2012 ▶), and the 25% threshold that we use to assess potential increases in the coverage based on future improvements in homology-modeling methods. We note that *UClust* generates clusters of proteins that are similar above the predefined threshold to a reference (seed) protein in a given cluster and that it defines similarity as the number of identical residues in the alignment divided by the length of the shorter sequence. This means that proteins within a given cluster are likely to have a pairwise sequence similarity above the threshold, although this is not guaranteed. We use this clustering method since it provides a good trade-off between the quality of clustering and low computational cost (Edgar, 2010 ▶), which is necessary given the large size of our UniProt data set. Using the clustering at 30% sequence identity, each protein sequence in a given cluster is considered ‘structurally covered’ at a given cutoff of the crystallization propensity score if there is at least one sequence in this cluster with a score higher or equal to the cutoff. The structures of the remaining sequences in that cluster could be obtained through homology modeling. These clusters are referred to as ‘modeling families’. Moreover, the percentage values of coverage are computed with respect to the total number of modeling families in a given analysis, *i.e.* the number of structurally solved modeling families divided by the total number of modeling families.

To estimate the current coverage by X-ray structures, we used the *USearch* algorithm to map proteins from the UniProt data set to the PDB data set. More specifically, we found all proteins from the UniProt data set which have at least one target in the PDB data set which covers no less than 90% of their sequence with no less than 90% sequence identity. As above, we assume that a given cluster (modeling family) can be solved by homology modeling if at least one of its members has such a PDB target, *i.e.* if a template structure for homology modeling is already available in PDB.

Supplementary Table S2 summarizes the scope of our study, including the number of considered complete proteomes, protein sequences and modeling families across three superkingdoms and viruses.

### 2.3. Measures to evaluate predictive quality   

To estimate a correlation of inputs (features) used by our predictors with the binary prediction outcome (crystallizable *versus* noncrystallizable), we use the point biserial correlation coefficient, 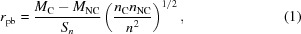
where *S_n_* is the standard deviation of the values of a given feature on the entire data set of proteins (both crystallizable and noncrystallizable), *M*_C_ and *M*_NC_ are the mean values for the crystallizable and noncrystallizable proteins, respectively, *n*_C_ and *n*_NC_ are the numbers of crystallizable and noncrystallizable proteins, respectively, and *n* is the total number of proteins.

The predictive quality of the crystallization propensity predictors was evaluated utilizing several commonly used measures including accuracy, sensitivity, specificity and the Matthews correlation coefficient (MCC) (Overton & Barton, 2006 ▶; Overton *et al.*, 2008 ▶, 2011 ▶; Kurgan *et al.*, 2009 ▶; Kanda­swamy *et al.*, 2010 ▶; Mizianty & Kurgan, 2011 ▶, 2012 ▶; Charoen­kwan *et al.*, 2013 ▶). Receiver operating characteristic (ROC) curves are typically used to evaluate the numerical propensity scores associated with the predictions of binary outcomes. Using each unique propensity value generated by a given classifier as a threshold, all predictions with scores that are equal or greater than a given threshold are set as the predicted positives (crystallizable proteins) and all other proteins are set as the predicted negatives (noncrystallizable proteins). Next, the TP rate and FP rate are calculated and plotted on a two-dimensional graph to form the ROC curve. We compute the area under the ROC curve (AUC) to quantify the predictive quality. Higher AUC values correspond to higher quality of predictions.

### 2.4. Design of *fDETECT*   

We used a machine-learning approach and annotated (with the prediction outcomes) data from the training data set to build the *fDETECT* prediction model. Our model predicts whether a given input protein chain would yield or fail to yield a high-quality crystal structure. It also outputs the propensity of crystallization, which is higher for chains that are predicted to crystallize and lower for chains that are predicted to fail to crystallize. We converted each amino-acid sequence into a fixed-sized array of numerical features that represent various physicochemical characteristics of this chain. These features are inputted into the *fDETECT* model to generate the predictions; the model cannot directly use the protein chain as the input since it has variable size. We considered a comprehensive set of features and empirically selected and used a subset of relevant (to the prediction of crystallization propensity) subset of features utilizing the training data set. We also evaluated several machine-learning algorithms on the training data set to select the model that provides the highest predictive performance.

#### 2.4.1. Features   

We considered a comprehensive set of features when designing our predictor. In total, we analyzed 1276 features that were computed utilizing amino-acid indices from the AAIndex database (Kawashima *et al.*, 2008 ▶), motivated by the work described in Mizianty & Kurgan (2011 ▶), protein characteristics such as absorption, instability, net charge, extinction and isoelectric point, amino-acid groupings based on their physicochemical properties, disorder predictions with the *IUPred* method (Dosztányi *et al.*, 2005 ▶) and predictions of low-complexity regions (Wootton & Federhen, 1993 ▶). The designed features cover global protein properties (*e.g.* the average value of the index over the whole protein sequence and the number of sequence segments with a given characteristic) as well as local characteristics (*e.g.* the maximal/minimal value of an index over all sliding segments composed of consecutive residues). The list of features with detailed descriptions is available in §S1 of the Supporting Information.

#### 2.4.2. Feature selection   

Feature selection was implemented using two steps: (i) the removal of irrelevant and redundant features and (ii) wrapper-based feature selection which selects a subset of remaining features to maximize the predictive performance on the training data set.

In the first step, the 1276 considered features were filtered to remove features that have weak or no correlation with the predictive outcome and which are cross-correlated with each other. We removed features with a biserial correlation with the outcome (crystallizable *versus* noncrystallizable) lower than double the value of the average biserial correlation in the set of all considered features. Removal of cross-correlated features produced a subset of the remaining features for which all possible pairs of features have Pearson correlations below 0.7 (58 features) and below 0.3 (11 features), which are utilized in the second step to perform wrapper feature selection and the parameterization of classifiers utilized in the wrappers, respectively.

In the second step, we considered three popular types of classifiers: support vector machine (SVM), logistic regression with ridge estimator and normalized Gaussian radial basis function (RBF) network. For the SVM classifiers we considered linear, polynomial, RBF and sigmoid kernels. Each classifier (and kernel in the case of SVM) was parameterized, using the 11 features selected in the previous step, in order to maximize the AUC measure on the training data set. Using each of these optimized classifiers, we performed feature selection with the 58 features. Starting with the feature with the highest biserial correlation, we kept adding the subsequently ranked, according to the biserial correlation, features to the selected set of features if the addition of a given feature improved the AUC score.

These two steps were performed using fivefold cross-validation on the training data set. The correlations of features were computed as average values over the five training folds, and the AUC values of the classifiers were computed as averages over the five test folds. We maximized the AUC score that evaluates predicted propensities.

We evaluated all considered setups based on their predictive quality and their runtime; the results are presented in Supplementary Table S3. The difference between the best and worst AUCs was only 0.04. We selected the logistic classifier with 11 features to implement *fDETECT* since this design is three orders of magnitude faster than the best-performing setup and its AUC score was worse only by 0.001. The set of 11 features that were empirically selected and used in *fDETECT* is discussed in §S2 of the Supporting Information. We demonstrate that these features are well grounded in the literature and have been shown to be markers of crystallization outcomes. Our study formulates a novel combination of these characteristics that can be calculated quickly and which offers competitive levels of predictive performance for the prediction of crystallization propensity.

### 2.5. Evaluation   

We evaluated *fDETECT* on the test data set and compared it with a comprehensive set of existing methods including *OB-Score* (Overton & Barton, 2006 ▶), *XtalPred* (Slabinski, Jaroszewski, Rodrigues *et al.*, 2007 ▶; Slabinski, Jaroszewski, Rychlewski *et al.*, 2007 ▶), *CRYSTALP*2 (Kurgan *et al.*, 2009 ▶), *MetaPPCP* (Mizianty & Kurgan, 2009 ▶), *SVMCrys* (Kanda­swamy *et al.*, 2010 ▶) and *PPCPred* (Mizianty & Kurgan, 2011 ▶). The results given in Table 1 ▶ show that *fDETECT* gave the highest AUC score and the second best accuracy and MCC. The analysis reveals that *fDETECT*, while having comparable predictive performance to the best-performing *PPCpred*, is six orders of magnitude faster than this method and the next best predictor *XtalPred*. Although *fDETECT* is slightly slower than *CRYSTALP*2, it gives accuracy, MCC and AUC values which are significantly higher.

We also evaluated *fDETECT* on the PDB data set. Interestingly, our analysis reveals a trend between the resolution of the crystal structures and our predicted propensity. A higher score on average indicates that the corresponding structure has better resolution (Supplementary Fig. S1).

Our method was also validated by showing that the average crystallization propensities computed for chains with structures in the PDB (resolution < 3.5 Å) are higher than the average scores for all chains from the same proteomes (Supplementary Fig. S2). The positive value of the relative difference denotes that the scores for the PDB structures are higher than for all chains from the same proteomes. We observe two trends: (i) the relative differences are lower for bacterial proteomes, which overall have a high propensity for crystallization, and (ii) the relative differences are high for eukaryotes, *i.e.* the already solved structures have a substantially higher propensity for crystallization compared with the overall propensity; this means that the chains that remain to be solved are harder to crystallize.

### 2.6. Normal skewed distribution fitting   

We fitted a normal skewed distribution to model the distribution of median crystallization scores for three superkingdoms (Supplementary Table S4). We used the SN package for the R language to fit and generate the distributions.

## 3. Results   

Utilizing *fDETECT*, we analyzed crystallization propensity for a snapshot of the protein universe which consists of 8 652 940 nonredundant proteins encoded in 1953 complete proteomes (106 archaea, 1043 bacteria, 265 eukaryotes and 539 viruses) available in the 2012_07 release of the UniProt database (details of protein/species distribution are given in Supplementary Table S2). Our analysis aims to reveal the maximum attainable coverage by X-ray structures, which combines the current protocols for crystallization-based structure determination (through the use of crystallization propensity) and homology modeling. The majority of the analysis, unless otherwise stated, was performed on the protein data sets clustered at 30% sequence identity, a threshold which is typically considered as the minimum for accurate homology modeling (Baker & Sali, 2001 ▶; Nair *et al.*, 2009 ▶; Gront *et al.*, 2012 ▶). Each protein sequence cluster is considered to be ‘structurally covered’ at a given cutoff of the crystallization propensity score if there is at least one sequence in this cluster with a score higher or equal to the cutoff; hence, all remaining protein structural models could be obtained through homology modeling. We refer to these clusters as ‘modeling families’.

We divide the proteomes into three superkingdoms of life, archaea, bacteria and eukarya, and separate viral proteins into archaeal, bacterial and eukaryotic viruses; the latter was motivated by substantial differences in their crystallization propensities. Moreover, we include propensity data for two eukaryotic organelles: chloroplasts and mitochondria. We also estimate the coverage by X-ray structures across all GO annotations including molecular functions, biological processes and cellular components. Finally, we present the results for two case studies of high biological interest and biomedical impact: the *Homo sapiens* proteome and G-protein-coupled receptors (GPCRs), a family of transmembrane proteins. The *H. sapiens* proteome analysis reveals the current level of coverage by X-ray structures and provides an estimate of the attainable coverage using current crystallization and structure-determination protocols and homology-modeling algorithms. The analysis of GPCRs not only confirms that this family of proteins is highly challenging to crystallize, but also shows a possible application of our method in target selection as it is able to identify targets which are likely to be more suitable for crystallization. We chose GPCRs for our case study since this is a biomedically important family of membrane proteins involved in cellular signalling that encodes roughly 21% of the genes of known function (Roth, 2005 ▶; Schwartz & Hubbell, 2008 ▶) and represents 50–60% of current drug targets (Lundstrom, 2009 ▶). The numbers of considered complete proteomes, proteins, modeling families and GO annotations are summarized in Supplementary Table S2.

### 3.1. Attainable coverage by X-ray structures of modeling families   

**Figure 1:**
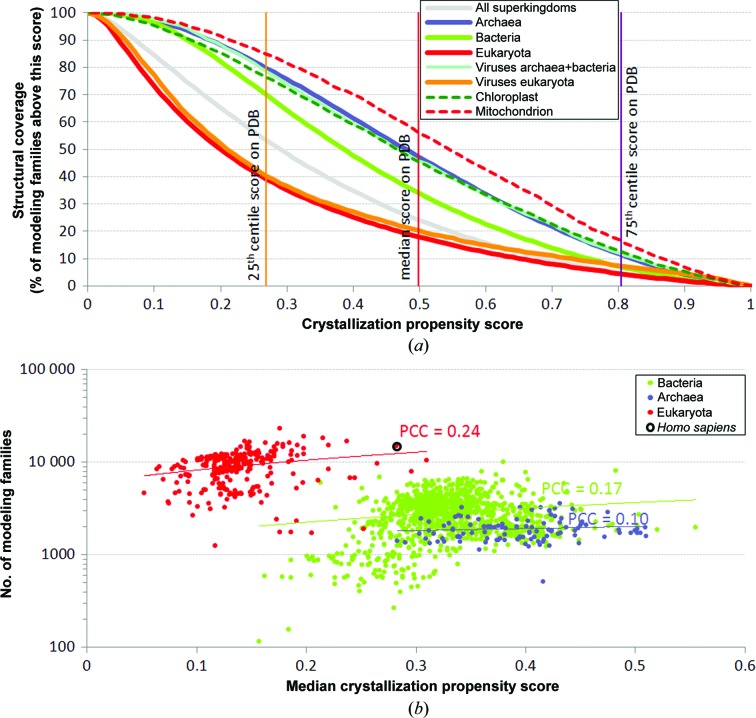
The coverage that is attainable by combining X-ray crystallography and homology modeling for modeling families. (*a*) The relationship between the crystallization propensity score and the corresponding coverage by X-ray structures for modeling families (*i.e.* a given modeling family is considered as having a structural model when any of its members has a crystallization score above a given cutoff) in a combined set of all considered complete proteomes, in eukaryotes, bacteria, archaea and viruses and in proteins located in chloroplasts and mitochondria. The vertical lines show the cutoff values that correspond to the 25th centile, the median and the 75th centile of the crystallization propensity scores of the clustered proteins from the PDB data set. (*b*) Scatter plot of the median propensity scores of complete proteomes grouped by their superkingdoms against the corresponding number of modeling families (*y* axis on a logarithmic scale). The scatter for each superkingdom was linearly fitted (thin line) and the corresponding Pearson correlation coefficient (PCC) is shown. Smaller proteomes (<100 modeling families) and viruses that also mostly include small proteomes were excluded to assure statistically sound estimates of propensities.

There are 1 734 048 modeling families in the set of 8 652 940 nonredundant proteins present in the 1953 complete proteomes. Fig. 1 ▶(*a*), which summarizes the coverage that is attainable by combining X-ray crystallography and homology modeling, shows that the three superkingdoms have a very different overall propensity for crystallization. Archaeal proteomes have the highest propensity (these proteins are the easiest to crystallize), eukaryotic proteomes have the lowest (the hardest to crystallize) and bacterial proteomes fall in between. Fig. 1 ▶(*a*) also reveals that organisms in each superkingdom express a large fraction of proteins that have high crystallization propensity scores and should be relatively easy to crystallize. We evaluated the propensity scores for a representative set of 50 138 nonredundant protein chains that have high-quality structures in the PDB (see the ‘PDB data set’ in §[Sec sec2]2) to define a cutoff score that corresponds to a high likelihood of proteins being successfully crystallized and producing structures. We clustered these proteins at 30% sequence identity to avoid bias towards folds that are overrepresented in the PDB and computed the cutoff value as the median over the average scores computed per cluster, which equals 0.498. This value is similar to the median value of the propensity for the crystallizable proteins in the benchmark test set used to evaluate *fDETECT*, which was 0.45. Assuming that proteins with scores higher than 0.498 are likely to be solvable *via* X-ray crystallography, the corresponding coverage by the X-ray structures varies by superkingdom and ranges from 19% of the modeling families in eukaryotes to 49% for archaea, with an overall coverage over all superkingdoms of 25%. We note that the coverage values are substantially higher (40% for eukaryota, 81% for archaea and 55% overall) if we assume that modeling families with members that have a propensity above the 25th centile of the propensities of proteins in the PDB, which is 0.268, would be solvable. Interestingly, proteins encoded by viruses show two distinct distributions. Viruses that infect eukaryotic organisms contain proteins with propensities similar to those of their host organisms. Viruses that infect bacteria or archaea have higher crystallization propensity values that are similar to the propensities of archaeal proteins. Moreover, proteins from mitochondria and chloroplast organelles, which are believed to have evolved from engulfed prokaryotes that once lived as independent organisms, show a high propensity similar to those of archaea and bacteriophages. Supplementary Fig. S3 shows the same analysis but considering all proteins instead of the modeling families. Although the relative differences in the coverage over different superkingdoms are similar, the values of the coverage are lower and are 6% for eukaryota, 31% for archaea and 14% overall. This suggests that homology modeling provides a substantial contribution towards the coverage, *i.e.* a modeling family is covered if at least one member can be solved and the structures of the remaining members are assumed to be predictable.

Fig. 1 ▶(*b*) demonstrates that eukaryotic organisms have larger complete proteomes, as measured by their number of modeling families. It suggests that as the number of substantially different protein sequences (*i.e.* modeling families) expands in these genomes their crystallization propensity becomes higher on average. This is confirmed by a modest (0.24) correlation between the number of modeling families in eukaryotes and their crystallization propensity.

### 3.2. Propensity for crystallization of complete proteomes   

**Figure 2:**
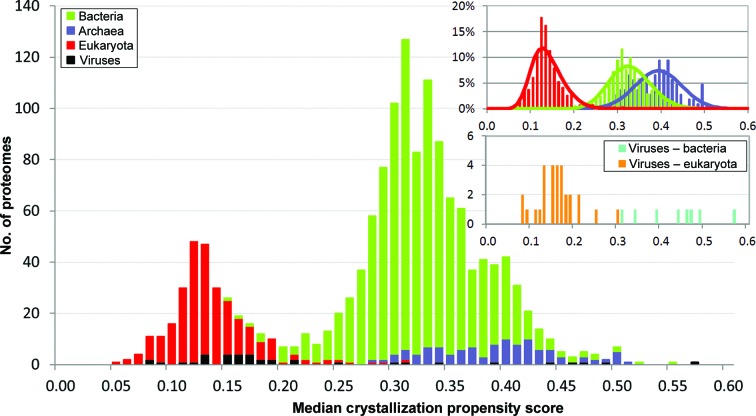
Histograms of the median crystallization propensities of the considered 1486 complete proteomes. Median crystallization propensities were computed across modeling families in each proteome assuming that a given family is represented by the highest score of its members. Modeling families were computed at the individual proteome level to exclude bias from the presence of sequence orthologs in other proteomes. The *x* axis is binned in intervals of 0.02 in width. The insets in the upper right corner show histograms for each of the three superkingdoms and fitted normal skewed distributions (lines), except for viruses, for which there are too few data points to fit the distribution. Smaller proteomes (<100 modeling families), which includes all proteomes of viruses that infect archaea, were excluded to assure statistically sound estimates of propensities.

We calculated the crystallization propensities of a subset of 1486 complete proteomes that have at least 100 modeling families (Supplementary Table S2); each proteome is represented by its median crystallization propensity score across its modeling families (Fig. 2 ▶). Similar to the results in Fig. 1 ▶(*b*), the crystallization propensities of the complete proteomes grouped by the three superkingdoms form different and distinct distributions; however, further details emerge. The bacterial and archaeal superkingdoms have proteomes with the broadest histograms of scores and with relatively high and low propensities. The propensities of bacterial proteomes overlap with those of archaeal and, to a smaller extent, eukaryotic proteomes. The propensities for archaeal and eukaryotic proteomes show almost no overlap. The histograms are bell-shaped and can be fitted by normal distributions (see inset in Fig. 2 ▶). Our results suggest that each superkingdom includes proteomes that may be easier or harder to crystallize. As suggested in Fig. 1 ▶(*a*), viruses show a ‘bimodal’ histogram and map into two distinct clusters (see the inset in Fig. 2 ▶). Eukaryotic viruses have proteomes with eukaryote-like propensities and bacteriophages have propensities that are similar to those of archaeal and bacterial proteomes (the number of modeling families in archaeal viruses is small and thus they were excluded from this analysis). Therefore, it appears that the crystallization propensities of viral proteomes co-evolved with and show properties that are similar to the host proteomes in their respective superkingdom.

### 3.3. Attainable coverage by X-ray structures of complete proteomes   

**Figure 3:**
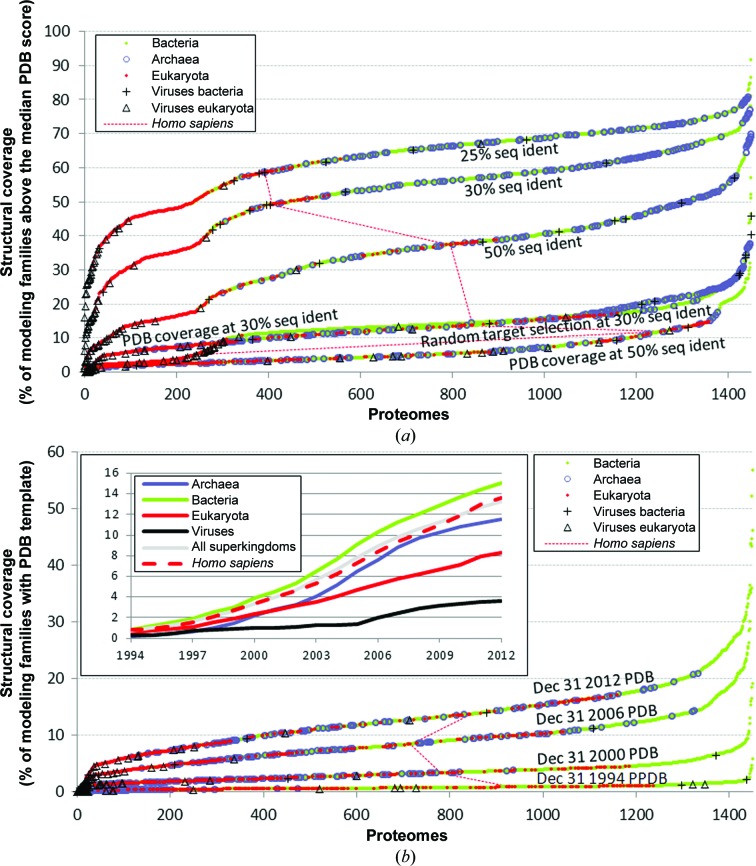
Coverage by X-ray structures for the considered 1486 complete proteomes grouped into the three superkingdoms of life and viruses. (*a*) The current coverage (‘PDB coverage’ plots) and the attainable coverage by combining X-ray crystallography and homology modeling (the remaining plots) of individual proteomes (shown using points) which are grouped into lines depending on the specific criteria used. The *x* axis lists all considered proteomes that are sorted based on their coverage by X-­ray structures; colors/markers of points indicate their taxonomic category. The coverage quantifies the fraction of modeling families in a given proteome that currently are or can be structurally solved. A given modeling family can be structurally covered if it includes at least one protein with a crystallization propensity above the median propensity of the clustered proteins from the PDB; the remaining structures in that family can be obtained using homology modeling. The top two lines show the coverage when modeling families are established based on different levels of sequence identity (25 and 30%); 30% corresponds to the current limits of homology modeling. The ‘50% seq ident’ line is used to analyze proteins families that share similar functions. The line labeled ‘random target selection’ shows the coverage by X-ray structures where targets in a given modeling family are selected at random instead of using the chain with the highest crystallization propensity. The two lines labeled ‘PDB coverage’ refer to the actual (current) coverage based on homology modeling (assuming the ability to predict structures at 30 or 50% identity) using existing structures in the PDB as templates. The dotted red line indicates the position of the human proteome. Smaller proteomes (<100 modeling families) were excluded to assure statistically sound estimates of propensities. (*b*) Changes in the coverage by X-ray structures over time. The four lines labeled with dates refer to the actual coverage based on homology modeling (assuming the ability to predict structures at 30% identity) using structures available in the PDB at a given time as templates. The inset shows the growth of average coverage aggregated for all considered proteins, each superkingdom, viruses and human proteins.

Fig. 3 ▶(*a*) plots the attainable coverage by combining X-ray crystallography and homology modeling of modeling families in complete proteomes using several scenarios. As we discussed earlier, we assume that a given modeling family is covered by X-ray structure(s) if it includes at least one protein with a crystallization propensity above the median propensity of clustered proteins from the PDB data set (proteins with such high scores are likely to be solvable *via* X-ray crystallo­graphy); the remaining structures in that family can be obtained using homology modeling. The results for 1486 complete proteomes that have over 100 modeling families are compared and grouped into the three superkingdoms and viruses. The ‘PDB coverage’ plot shows the coverage by X-ray structures of these proteomes (each point corresponds to one complete proteome) using structures currently available in the PDB and homology modeling. The ‘random target selection’ plot shows the attainable coverage by X-ray structures when protein sequences are selected at random for structure determination instead of selecting the chains with the best crystallization propensity scores. This is intended to represent the coverage that could be achieved using a more traditional way of selecting protein targets when the crystallization propensity score is not used to prioritize targets for structure determination but instead an arbitrarily chosen chain is solved. The top two plots in Fig. 3 ▶(*a*) show the coverage by X-ray structures that can be obtained when structures of all solvable modeling families (*i.e.* with scores above the median score for the PDB structures clustered at 30% sequence identity) are available and homology modeling is used to generate additional models using different sequence-identity cutoffs. With these assumptions and the 30% sequence-identity homology-modeling cutoff, virtually all bacterial and archaeal proteomes as well as bacterial viruses can be structurally covered by X-ray structures at above 50%. However, the majority of eukaryotes and eukaryotic viruses show coverage substantially below 50%. There is a visible decline in coverage between bacterial, bacterial virus and archaeal proteomes and the lower part of individual plots (eukaryotic viruses and eukaryotes). To account for the projected improvements in homology modeling, proteomes were clustered at a 25% sequence-identity cutoff. The coverage by X-ray structures increases by a substantial margin, on average about 10%, with the improved homology modeling for proteomes from all superkingdoms. Assuming that homology modeling would generate high-quality structures at 25% sequence identity, which should be possible based on the analysis of Gront *et al.* (2012 ▶), the coverage increases to over 60% of modeling families for most bacteria and archaea and to over 40% for most proteomes, except for some eukaryotes and eukaryotic viruses, for which the coverage would be >20%. We also considered clustering proteins at the 50% sequence-identity cutoff; the function of proteins is conserved at this sequence identity (Addou *et al.*, 2009 ▶; Rentzsch & Orengo, 2013 ▶). We observe that the corresponding attainable coverage by X-ray structures of most eukaryotes is fairly low (below 25%); however, some eukaryotic organisms (red points in the ‘50% seq ident’ line in Fig. 3 ▶ *a*), including human, have modest levels of coverage of close to 40%.

Fig. 3 ▶(*b*) shows how the coverage by X-ray structures has changed over time. We observe a marked difference in the coverage values of archaeal proteomes, which in the early days were among the lowest and have substantially improved over the last decade. In contrast, the coverages of eukaryotes and bacteria remain at the same levels relative to each other. We also note a substantial increase in coverage after the year 2000, which coincides with the creation of the Protein Structure Initiative (PSI). Interestingly, although the coverage by X-ray structures of eukaryotic organisms remains lower than for the other two superkingdoms, the human proteome has enjoyed relatively higher improvements in coverage, especially in the last decade (see inset in Fig. 3 ▶ *b*). This is a remarkable achievement given that human proteins are substantially harder to solve *via* X-ray crystallography compared with proteins that have already been structurally solved (see §[Sec sec3.5]3.5).

These results demonstrate that major technological advances in experimental protein structure determination will be needed to make a greater impact on the coverage. They also show that the use of target selection and prioritization based on the crystallizability propensity score allows coverage by the X-ray structures to be substantially improved by 25–40% and makes structural models available to the community for more protein families.

### 3.4. Coverage by the X-ray structures of GO functional annotations   

**Figure 4:**
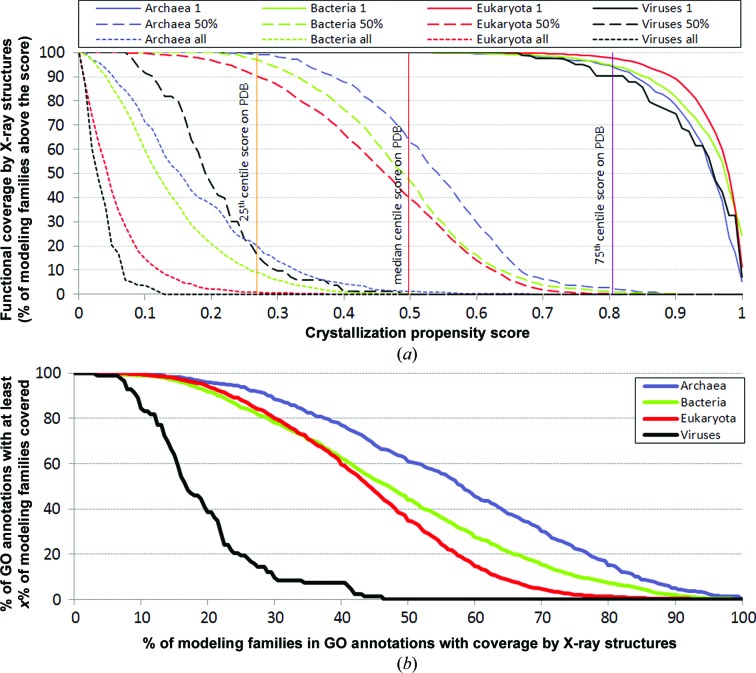
Functional coverage (fraction of structural families with given GO annotations that can be solved with X-ray structures) of the considered 4719 GO annotations across the three superkingdoms of life and viruses. Proteins with given GO annotations were mapped into modeling families. (*a*) Results on assuming that a GO annotation is covered when a given fraction of its structural families is solved. The solid lines assume that a given GO annotation is covered when one or more of its annotated modeling families has an obtainable structure. The dashed/dotted lines assume that a given annotation is covered when at least 50%/all of its modeling families are structurally covered. The vertical lines show the cutoff values that correspond to the 25th centile, the median and the 75th centile of the crystallization propensity scores of the clustered proteins from the PDB data set. (*b*) Analysis of how many GO annotations in a given superkingdom (*y* axis) have at least a given fraction of modeling families amenable to structure solustion (*x* axis). We assume that a given modeling family can be structurally covered if it includes at least one protein with a crystallization propensity above a cutoff value provided on the *x* axis in (*a*) or above the median score (0.498) for the PDB structures clustered at 30% sequence identity in (*b*); the remaining structures in that family can be obtained using homology modeling. To assure statistically sound estimates and to accommodate for the incompleteness of the GO annotations we limited analysis to annotations with at least 20 modeling families.

An important issue to consider is how many structures of proteins with different functions can be obtained through X-ray crystallography and computational approaches. We calculated the coverage by the X-ray structures of functions, as represented by GO annotations, for the 1953 complete proteomes. Each GO annotation is represented by a set of proteins with this annotation. To accommodate the added value of homology modeling, we clustered the UniProt data set at 30% sequence identity to define modeling families and we mapped annotated proteins to these clusters. In the 2012_07 release of the UniProt database that we utilized there are 4 960 913 non­redundant proteins with 4719 unique GO annotations that have at least 20 modeling families (57% of all considered protein sequences); a detailed breakdown of the number of considered GO annotations is given in Supplementary Table S2. The reason for limiting our analysis to annotations with higher counts is to accommodate the incompleteness of the GO annotations and to produce statistically sound estimates. Assuming that a given annotation has at least one of its structures available based on the attainable coverage that combines X-ray crystallography (the crystallization propensity score is above the median score of the clustered PDB structures) and homology modeling (using a 30% sequence-identity cutoff to provide the predicted structure), virtually all annotations can be structurally covered across all superkingdoms (see solid lines in Fig. 4 ▶ *a*).

However, when considering that a given GO annotation is structurally covered when at least half of its modeling families have a structure, the coverage varies considerably between superkingdoms, with 40% for eukaryotes, 47% for bacteria, 63% for archaea and no coverage for viruses (see dashed lines in Fig. 4 ▶ *a*). Fig. 4 ▶(*a*) also demonstrates that it would be unlikely that structures would be obtained for all modeling families in each GO annotation; the corresponding coverage by the X-ray structures approaches 0% for bacteria, eukary­otes and viruses, and is only about 1% for archaea (Fig. 4 ▶ *a*, dotted lines). This is because most of the GO annotations include modeling families with a low crystallization propensity score that may be very hard to crystallize and determine a structure. Among the most challenging functional families are membrane proteins such as ryanodine-sensitive calcium-release channels, voltage-gated calcium channels and G-protein-coupled acetylcholine receptors.

In Fig. 4 ▶(*b*) we show how many GO annotations (*y* axis) have at least a given fraction of modeling families amenable to solution of their structures (*x* axis). These data are analyzed for each superkingdom of life and viruses. For instance, for eukaryotic species by following the red line we observe that 80% of GO annotations (*y* axis) have at least 30% of their modeling families (*x* axis) solvable and only 20% of the annotations (*y* axis) have at least 58% of the families amenable to structure determination.

The results can be divided by a particular type of annotation, such as cellular components, molecular functions and biological processes (Supplementary Figure S4). The attainable coverage by the X-ray structures values per annotation type follow the same trends as in Fig. 4 ▶(*a*) that combines all GO functional annotations.

### 3.5. Analysis of the human proteome   

Obtaining structures or accurate homology models for human proteins is clearly of high importance. The results of the analysis of the complete *H. sapiens* proteome are summarized in Figs. 3 ▶(*a*) and 5 ▶. The values of the current and the attainable coverage by X-ray structures for the complete *H. sapiens* proteome given in Fig. 3 ▶(*a*) (dotted red lines) are among the highest in the eukaryotic proteomes. We estimate the current coverage (based on PDB structures and homology modeling) to be 14% of modeling families and 26% of proteins, *i.e.* the already covered modeling families are relatively large. Out of 70 101 human proteins, 6603 have structures in the PDB and a further 11 804 can be predicted by homology modeling. To compare, recent work that collected human protein structures (using PDB depositions and predicted structures) to predict protein–protein interactions reported a similar coverage of 28% (Zhang *et al.*, 2012 ▶). Fig. 3 ▶(*a*) reveals that the coverage can be substantially improved to 49% of modeling families assuming that all human proteins with high crystallization propensities (above the median score computed for the PDB structures) will be solved and the corresponding modeling families will be predicted *via* homology modeling. Assuming that homology modeling would be successful at 25% sequence identity, the coverage would further increase to 59%. Interestingly, solving the structures of human proteins selected at random from each modeling family would lead to an estimated coverage of 5% of the modeling families, which is lower than the current coverage. This is owing to the fact that proteins in *H. sapiens* are relatively difficult to crystallize; their median crystallization propensity is 0.28 compared with a median score for the clustered PDB structures of 0.498.

**Figure 5:**
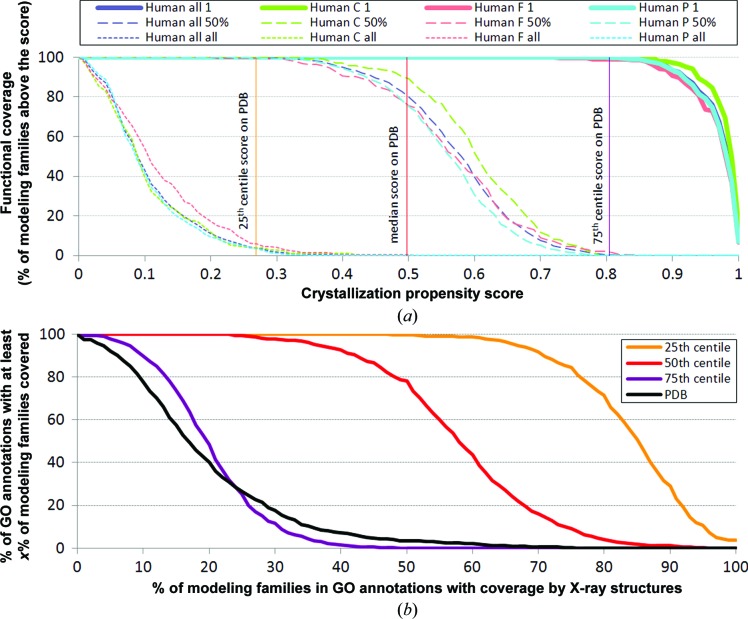
Coverage by X-ray structures of GO annotations in the *H. sapiens* proteome. (*a*) Functional coverage of the *H. sapiens* proteome (number of GO annotations with X-ray structures divided by the number of all available GO annotations in *H. sapiens*). The colors of the lines correspond to the results for all GO annotation types (all) and for biological processes (P), molecular functions (F) and cellular components (C) annotations. Human proteins with given GO annotations were mapped into modeling families. A given modeling family can be structurally covered if it includes at least one protein with a crystallization propensity above the cutoff value provided on the *x* axis; the remaining structures in that family can be obtained using homology modeling. The solid lines assume that a given GO annotation is covered when one or more of its annotated modeling families has an obtainable structure. The dashed/dotted lines assume that a given annotation is covered when at least 50%/all of its modeling families are structurally covered. The vertical lines show the cutoff values that correspond to the 25th centile, the median and the 75th centile of the crystallization propensity scores of the clustered proteins from the PDB data set. To assure statistically sound estimates we limited analysis to the annotations with at least 20 modeling families. (*b*) The current (black line) and the attainable (violet, red and yellow lines) coverage by X-ray structures of the annotated proteins in the complete *H. sapiens* proteome. The *y* axis shows the percentage of annotations which have at least *x*% of their modeling families covered, where the value of *x* is given on the *x* axis. Lines labeled as the 25th, 50th and 75th centiles are the coverage by X-ray structures when we assume that a given protein can be solved if its score is higher than the the 25th, the 50th (median) and the 75th centile, respectively, of propensity scores of the clustered structures from the PDB data set.

The coverage by X-ray structures of GO functional annotations in human proteins is presented in Fig. 5 ▶(*a*), whereas Fig. 5 ▶(*b*) compares the actual (based on structures presently available from the PDB) and the attainable coverage values for these GO annotations. Assuming that a given GO annotation is structurally covered if at least one of its proteins has an attainable structure (by combining X-ray crystallography and homology modeling at 30% sequence identity), one can structurally cover virtually all annotations in the human proteome (Fig. 5 ▶ *a*). This is also consistent with Fig. 4 ▶(*a*) (‘Eukaryota 1’ plot). The coverage slightly decreases to about 80% overall if one assumes that at least half of the modeling families for a given annotation must have a structural model. However, the coverage decreases to nearly 0% if one wants to fully cover each annotation (*i.e.* to generate structures for all of its modeling families). This suggests that for virtually all annotations in the human proteome there are modeling families whose structures will be very difficult to obtain. Taken together, the results on the human proteome indicate higher coverage than for a generic set of eukaryotic proteomes (see Fig. 4 ▶ *a*). In Fig. 5 ▶(*b*) we show that the current coverage of functional annotations in the human proteome (based on structures in the PDB) is fairly low. Following the black line, 90% of annotations (*y* axis) have a coverage of at least 6% (*x* axis), while only less than 10% of annotations (*y* axis) have a coverage of over 35% (*x* axis). However, using X-ray crystallography and homology modeling the coverage can be substantially improved. Based on the red line (*i.e.* using the cutoff that assumes that a structure can be obtained if the corresponding crystallization propensity is larger than a median propensity of the clustered structures from the PDB data set), 90% of annotations (*y* axis) would have at least 42% coverage (*x* axis) and 50% would have at least 58% coverage. These coverage values are again larger than the corresponding values computed over all eukaryotes (red line in Fig. 4 ▶ *b*).

### 3.6. Analysis of GPCRs   

**Figure 6:**
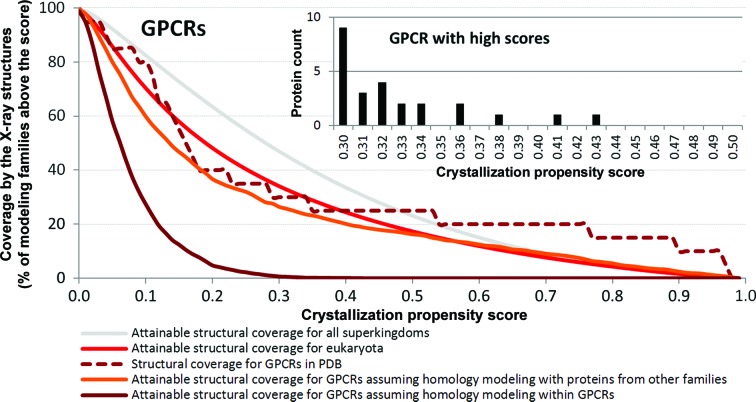
Coverage by X-ray structures of G-protein-coupled receptors (GPCRs). The *y* axis shows the percentage of annotations which have at least *x*% of modeling families covered, where the corresponding value of *x* is shown on the *x* axis. The dashed ‘GPCRs in PDB’ line shows the crystallization propensity scores for GPCRs that have been deposited in the PDB. A histogram of GPCRs with crystallization propensity scores above 0.3 is given in the inset.

24 730 members of the GPCR family were clustered at 30% identity and their attainable coverage by X-ray structures was analyzed per corresponding modeling family (Fig. 6 ▶). The coverage values of GPCRs (dark red line) are substantially lower than for eukaryotic families (red line) and all considered modeling families (gray line). GPCRs for which structures are already available in the PDB show relatively high crystallization propensity scores (dashed line) and this demonstrates that they could be identified and selected for structural studies using *fDETECT*. However, some of these structurally solved GPCRs are engineered protein fragments which may result in the production of protein chains with higher crystallization propensity scores. Assuming that GPCRs with crystallization propensities above the median propensity of clustered chains with structures in the PDB can be structurally solved, the combined use of X-ray crystallography and homology modeling that uses only GPCRs would fail to provide any structures of known modeling families of GPCRs (see the dark red line, crystallization propensity score of 0.498). The orange line, which shows the attainable coverage by the X-ray structures when homology modeling utilizes proteins from other protein families, shows a substantial improvement, with a coverage of 16.7%. We found some GPCR chains that have high propensities and their structures should be substantially easier to solve (see inset in Fig. 6 ▶). These 26 GPCRs with fairly high scores above 0.3 are given in Supplementary Table S5.

## 4. Discussion   

Although nature does not impose evolutionary pressure on protein sequences to improve their crystallization properties, it has been shown experimentally that many proteins can form ordered crystals that can be used to elucidate their atomic structures. Can we identify and rank these proteins and rationally select the best proteins for structural studies *via* X-ray crystallography? Our analysis shows that different completely sequenced proteomes vary substantially in their propensity for crystallization. It also appears that proteomes from the taxonomic superkingdoms have distinct crystallization propensities. These propensities can be fitted with a normal skewed distribution, where archaeal proteomes show the highest propensities (mean = 0.39), bacterial proteomes show intermediate values (mean = 0.33) and eukaryotic proteomes show the lowest scores (mean = 0.14) (Fig. 2 ▶; Supplementary Table S4). These distributions are broad and therefore depending on which organism is used to select the protein target it may be easier or more difficult to determine its structure. The differences in propensity for crystallization may illustrate proteome adaptation to environmental niches and highlight protein properties common to crystallization and adaptability. Archaea tend to occupy more extreme niches than bacteria and eukaryotes. These organisms are among the most thermophilic known to science and thus their structures are more stable, which is one of factors that correlates with high crystallization propensity (see the ‘Instability Index’ in Supplementary Fig. S5). In contrast, eukaryotes are characterized by a narrower thermal adaptation and many of their proteins show relatively high levels of intrinsic disorder (Ward *et al.*, 2004 ▶; Xue *et al.*, 2012 ▶; Peng *et al.*, 2014 ▶), which in turn makes the crystallization of these proteins more challenging (Oldfield *et al.*, 2013 ▶). Moreover, it seems that eukaryotic viruses co-evolved and have propensities that are very similar to their eukaryotic host proteomes and are also characterized by high adaptability and intrinsic disorder (Xue *et al.*, 2010 ▶). On the other hand, bacteriophages and archaeal viruses have propensities that are substantially higher and are similar to proteomes from the archaeal and bacterial superkingdoms. Proteins from mitochondria and chloroplast organelles, which are of prokaryotic origin, show a high crystallization propensity similar to archaeal and bacterio­phage proteomes.

As shown in Fig. 3 ▶(*a*), the coverage by X-ray structures of proteomes using the nonredundant structures currently available in the PDB is relatively low. The generation of additional structures by combining X-ray crystallography and improved homology modeling would considerably increase the coverage across individual proteomes and superkingdoms. The range of attainable coverage differs substantially between superkingdoms, being correspondingly higher for bacterial and archaeal proteomes and lower for eukaryotic proteomes and their viral proteins (Fig. 1 ▶ *a*). The use of more advanced homology-modeling methods that could provide additional accurate models at a lower sequence identity would lead to much higher coverage for proteomes of all superkingdoms and viruses (Fig. 3 ▶ *a*). The current coverage by X-ray structures based on the structures available in the PDB combined with homology modeling results in a similar distribution of coverage values between the superkingdoms and viruses compared with the distribution of the attainable coverage (*i.e.* all plots in Fig. 3 ▶ *a* have green and blue points on the right and red and triangle markers on the left); however, the values are consistently smaller. The main difference is a larger overlap between scores for the archaeal, bacterial and eukaryotic proteomes for the current coverage. Clearly, the X-ray structures that have been determined so far show a generally higher crystallization propensity score than proteins from all sequenced genomes (the median crystallizability propensity score for clustered proteins in the PDB is 0.498 compared with 0.23 for the considered genomes), which is expected because these proteins have been crystallized. However, these differences may not solely reflect crystallization propensity; they are possibly influenced by the research interests of individual laboratories and SG centers.

We also show that use of target selection methods based on estimation of crystallization propensity, such as our *fDETECT* method or other methods, can considerably increase the attainable coverage when compared with a ‘traditional’ approach that does not utilize such scores (Fig. 3 ▶ *a*). The ‘traditional’ approach is also associated with more pronounced differences in coverage values between superkingdoms (‘Random target selection’ plot in Fig. 3 ▶ *a*) compared with the rational approach (the top three plots in Fig. 3 ▶ *a*).

The analysis of the coverage by X-ray structures of functional and localization-based annotations defined in GO reveals that we can currently provide structural models for at least one protein in each functional annotation (Fig. 4 ▶ *a*). However, the fraction of annotations for which at least half of their modeling families or all modeling families can be crystallized varies widely between superkingdoms and is relatively low. This suggests that almost all of the annotations contain some very hard-to-crystallize proteins, and points out the necessity of developing new strategies for structure determination of these classes of protein families.

Inspection of the attainable coverage by X-ray structures in the *H. sapiens* proteome shows that it is one of the most structurally attainable proteomes among eukaryotes (Fig. 3 ▶ *a*). One of the reasons is that it attracts more attention and resources, resulting in more structures and more coverage. We also show that the coverage of GO annotations in humans can be greatly improved using ‘rational’ target selection and current crystallization and homology-modeling technologies (red *versus* black lines in Fig. 5 ▶ *b*).

Finally, we analyzed the idiosyncrasies of the coverage by X-ray structures for an important transmembrane protein family of GPCRs, which are found primarily in eukaryotes. Our study demonstrates that their crystallization propensity is relatively low. Nevertheless, we found that use of homology modeling could substantially increase the coverage by X-ray structures of this protein family. We also investigated the crystallization propensities of GPCRs for which structures have been deposited in the PDB and found that, as expected, their scores are higher compared with the overall scores for all GPCRs (Fig. 6 ▶). This means that so far easier GPCR targets have been crystallized and their structures solved, and that significant effort may be needed to determine the remaining GPCR targets. This result is also influenced by the fact that some of these proteins were engineered to enhance their crystallization propensity. We provide a list of 26 GPCR targets with crystallization propensity scores of at least 0.3; we believe that the structure of these proteins should be easier to crystallize.

Significant structural biology efforts on human proteins have contributed to one of the highest coverage values of this organism among all eukaryotes. However, our analysis reveals that current X-ray crystallographic knowhow can only determine a relatively small fraction of protein structures, particularly from viruses and most of the remaining eukaryotic proteomes. Many of these proteins have significant biomedical impact and are targeted for structure determination (for example GPCRs, HIV, SARS and influenza virus). Our data suggest that we need to continue major technological advances in experimental protein structure determination using X-ray crystallography to determine the structures of the most challenging proteins in order to make a greater impact on the coverage and to determine the structures of proteins that are targeted because their structures are important to understand a given disease and/or for drug discovery. Our analysis also shows that the use of ‘rational’ target selection and prioritization based on the crystallization propensity scores allows coverage by the X-ray structures to be substantially improved (Slabinski, Jaroszewski, Rodrigues *et al.*, 2007 ▶; Babnigg & Joachimiak, 2010 ▶). As petascale and exascale computing become available to biological research in the near future and new template-based algorithms and protocols are developed (Moult *et al.*, 2011 ▶; Zhang, 2014 ▶), this avenue could provide substantial improvements in the overall attainable coverage.

We believe that our method has helped to advance our understanding of the coverage by X-ray structures of proteins and complete proteomes on a global scale. The method exclusively uses amino-acid sequence information and existing experimental crystallization knowhow from large-scale SG efforts. The data sets used to develop and benchmark our method (see §[Sec sec2]2) include proteins taken directly from the TargetDB resource without affinity tags. At present our method cannot be applied to predict the crystallization propensity of protein–protein and protein–nucleic acid complexes, and does not consider small molecules such as cofactors and ligands. Moreover, we utilize the sequences in their wild-type form, which means that we do not consider modifications (mutations, affinity tags, constructs *etc*.) that could change the crystallization propensity. Homology modeling is assumed to produce structures with sufficient quality at 30% sequence identity, which may not always be realistic.

## Supplementary Material

Supporting Information.. DOI: 10.1107/S1399004714019427/dz5333sup1.pdf

## Figures and Tables

**Table 1:** Comparison of *fDETECT* and other predictors of crystallization propensity on the test data set Results are sorted according to the AUC score; the best value for each measure is given in bold. Results are reported as average (avg) and standard deviation (std) values over 100 repetitions that utilize 50% of the test data-set data that were selected at random; + or in the sig columns denotes results that are statistically significantly worse or better than the corresponding result from *fDETECT* using a *p*-value of 0.05. The significance of differences was computed with the Student’s paired *t*-test if the distributions were normal or with the Wilcoxon test otherwise. Distribution type was verified using the AndersonDarling test.

	Runtime per protein (ms)			Accuracy (%)			MCC			Specificity (%)		Sensitivity (%)		AUC		
Method	avg	std	sig	avg	std	sig	avg	std	sig	avg	std	avg	std	avg	std	sig
*fDETECT*	0.8	0.0		70.6	0.8		0.354	0.017		75.8	0.8	60.3	1.5	**0.754**	0.009	
*PPCpred*	152912.9	1438.1	+	**71.8**	0.8		**0.361**	0.017		**79.7**	0.8	56.0	1.5	0.741	0.009	+
*XtalPred* †	70624.4	1008.6	+	53.3	0.9	+	0.248	0.016	+	36.0	1.0	**87.6**	1.1	0.665	0.011	+
*CRYSTALP*2	**0.3**	0.0		56.6	0.8	+	0.202	0.015	+	48.5	0.9	72.6	1.3	0.658	0.010	+
*SVMcrys*	153.3	0.7	+	56.5	0.8	+	0.223	0.017	+	46.5	1.0	76.5	1.4	0.615	0.009	+
*OBScore*	64	0.2	+	47.2	0.9	+	0.130	0.017	+	29.3	1.0	82.7	1.1	0.569	0.010	+
*ParCrys* ‡	N/A	N/A	N/A	48.3	0.8	+	0.105	0.016	+	34.5	0.9	75.9	1.1	0.557	0.010	+

† The *XtalPred* results were obtained from a web server; the time estimation may be inaccurate.

‡ *ParCrys* is available as a web server and we could not estimate its time efficiency.

## Footnotes

1 Supporting information has been deposited in the IUCr electronic archive (Reference: DZ5333).
