# Supplementary material for: Covering complete proteomes with X-ray structures: a current snapshot
Source: Acta Crystallogr D Biol Crystallogr. 2014 Oct 23;70(Pt 11):2781–93. doi: 10.1107/S1399004714019427 (PMC4220968; doi:10.1107/S1399004714019427)
Supplement: Supplementary file 1 [file d-70-02781-sup1.pdf]

# Supporting Information for “Covering the complete proteomes with X-ray structures - a current snapshot”

Authors

**Marcin J. Mizianty[a], Xiao Fan[a], Jing Yan[a], Eric Chalmers[a], Christopher Woloschuk[a], Andrzej Joachimiak[b]* and Lukasz Kurgan[a]***

[a]Electrical and Computer Engineering, University of Alberta, Edmonton, Alberta, T6G 2V4, Canada

[b]Midwest Center for Structural Genomics, Argonne National Laboratory, Lemont, IL, 60439, USA

Correspondence email: andrzejj@anl.gov; lkurgan@ece.ualberta.ca

## S1. Considered Features

We computed and evaluated total of 1276 features/inputs for fDETECT that include:

*Amino acid based* (420 features). Features based on amino acid (AA) types.

- AAComposition_{AA} –composition (count) of a given AA type {AA} divided by protein’s sequence length.(20 features)
- AAComposition_{AA}_{AA} – composition (count) of a given dipeptide {AA}_{AA} divided by protein’s sequence length. (400 features)

*Amino acid group based* (336 features). Features based on division of AAs into groups characterized by specific physicochemical properties, see Supplementary Table S6. The twenty AAs are divided into three groups for each of the seven different AA characteristics representing the main clusters of the AA indices of Tomii and Kanehisa (Tomii & Kaneshita, 1996) that were presented in (Dubchak *et al*., 1999).

- GRComposition_{Char}_{Gr} – Composition of AAs belonging to a given group in a given characteristic divided by protein’s sequence length. This feature is computed for each group {Gr} of each characteristic {Char} in Supplementary Table S6. (7 characteristics x 3 groups = 21 features)
- GRTransition_{Char}_{$Gr_1$-$Gr_2$ or $Gr_2$-$Gr_1$; $Gr_1$-$Gr_3$ or $Gr_3$-$Gr_1$;$Gr_2$-$Gr_3$ or $Gr_3$-$Gr_2$} – frequency of occurrence of transitions between groups for a given characteristic within the input protein. We sum AA pairs that transition between different groups and divide by protein’s sequence length minus 1. This feature is computed for each of the three possible

IMPORTANT: this document contains embedded data - to preserve data integrity, please ensure where possible that the IUCr Word tools (available from http://journals.iucr.org/services/docxtemplate/) are installed when editing this document.

transitions ($Gr_1$to $Gr_2$ or $Gr_2$ to $Gr_1$; $Gr_1$ to $Gr_3$ or $Gr_3$ to $Gr_1$; $Gr_2$ to $Gr_3$ or $Gr_3$ to $Gr_2$) for each group characteristic{Char} in Supplementary Table S6. (7 characteristics x 3 transitions per group = 21 features)

- GRDistribution_{Char}_{Gr}_{first,$25^{th}$%,$50^{th}$%,$75^{th}$%,last} – Position of occurrence of {first,$25^{th}$ %,$50^{th}$ %,$75^{th}$ %,last} residue belonging to a given group {Gr} for a given characteristic divided by protein's sequence length. This feature is computed for each group {Gr} of each characteristic {Char} in Supplementary Table S6. (7 characteristics x 3 groups x 5 position choices = 105 features)
- GRSegmentCount_{Char}_{Gr}_{1-5, 6-10, 11-15, >15} – Count of the number of short (1-5 residues)/medium (6-10 residues)/long (11-15 residues)/very long (over 15 residues) segments of AAs that are exclusively in a given group {Gr} for a given characteristic {Char} listed in Supplementary Table S6. These counts were normalized by the total number of segments (for that group) in the input protein chain. (7 characteristics x 3 groups x 4 segment sizes = 84 features)
- GRSegmentComposition_{Char}_{Gr}_{1-5, 6-10, 11-15, >15} – the number of AAs in the input protein sequence that are in short (1-5 residues)/medium (6-10 residues)/long (11-15 residues)/very long (over 15 residues) segments of AAs that are exclusively in a given group {Gr }for a given characteristic {Char} listed in Supplementary Table S6. These counts were normalized by the protein's sequence length. (7 characteristics x 3 groups x 4 segment sizes = 84 features)
- GRLongestSegment_{Char}_{Gr} – the length of the longest segment of AAs that are exclusively in a given group {Gr} for a given characteristic {Char} listed in Supplementary Table S6 divided by the protein's sequence length. This feature is computed for each group {Gr} of each characteristic {Char} in Supplementary Table S6. (7 characteristics x 3 groups = 21 features)

*Amino acid index based* (448 features). These features are based on per AA values of hydrophobicity and energy based indices collected from the AAIndex database3; see Supplementary Table S7 for the list of the considered indices:

- AAindex_{Index}_avg –average value of a given AA index {Index} over the whole input protein sequence. These features are computed for each index {Index} in Supplementary Table S7. (64 indices = 64 features)
- AAindex_{Index}_{min,max}_{5,10,15} –The minimal/maximal average value of a given AA index {Index} among all sliding windows of sizes 5, 10, and 15 over the input protein

chain. For chains shorter than a given window size, we use the window size equal the length of the sequence. (64 indices x 6 values per index = 384 features)

*Protein's properties based* (4 features). Features based on selected physicochemical properties of proteins:

- pI –The isoelectric point of the input protein. (1 feature)
- AliphaticIndex –The aliphatic index of a protein is defined as the relative volume occupied by aliphatic side chains (alanine, valine, isoleucine, and leucine). It is regarded as a positive factor for the increase of thermostability of globular proteins. The aliphatic index of a protein is calculated according to Ikai (Ikai, 1980). (1 feature)
- InstabilityIndex –The instability index provides an estimate of the stability of a given protein (Guruprasad *et al*., 1990). (1 feature)
- NetCharge – Net charge of a given protein. (1 feature)

*Disorder and complexity predictions based* (68 features). Features based on predictions of residues disorder performed by IUpred (Dosztanyi *et al*., 2005), which includes predictions of both Short (IUpred_S) and Long (IUpred_L) disorder segments, and based on assignment of sequence complexity utilizing SEG algorithm (Wootton & Federhen, 1993):

- PRprobability_{IUpredL, IUPredS}_avg – average value of probabilities/complexity values of a given predictor/algorithm {IUpredL, IUPredS, Complexity} over the whole protein sequence. (2 predictors = 2 features)
- PRprobability_{IUpredL, IUPredS}_{min,max}_{5,10,15} – The minimal/maximal average value of probabilities/complexity values of a given predictor/algorithm {IUpredL, IUPredS, Complexity} among all sliding windows of sizes 5, 10, and 15. For chains shorter than a given window size we use the window size equal the length of the sequence. (2 predictors x 6 values per index = 12 features)
- PRSegmentCount_{IUpredL, IUPredS, Complexity}_{0, 1}_{1-5, 6-10, 11-15, >15} – count of the number of short (1-5 residues)/medium (6-10 residues)/long (11-15 residues)/very long (over 15 residues) segments in the input protein for each binary prediction/complexity value {0, 1} of each predictor/algorithm{IUpredL, IUPredS, Complexity}. These counts were normalized by the total number of segments (for that predictor) in the protein. (3 predictors/algorithm x 2 predictions/assignments per predictor/algorithm x 4 segmentsizes = 24 features)

- PRSegmentComposition_{IUpredL, IUPredS, Complexity}_{0, 1}_{1-5, 6-10, 11-15, >15} – count of the number of AAs in the input protein sequence that are in short (1-5 residues)/medium (6-10 residues)/long (11-15 residues)/very long (over 15 residues) segments for each binary prediction/complexity value {0, 1} of each predictor/algorithm {IUpredL, IUPredS, Complexity}. These counts were normalized by the length of the protein. (3 predictors/algorithm x 2 predictions/assignment per predictor/algorithm x 4 segment sizes = 24 features)
- PRLongestSegment_{IUpredL, IUPredS, Complexity}_{0, 1} – the length of the longest segment for each binary prediction/complexity value {0, 1} of each predictor/algorithm{IUpredL, IUPredS, Complexity}divided by the protein sequence length.(3 predictors x 2 predictions per predictor = 6 features)

### S2. Features related to crystallization

fDETECT uses 11 features which are correlated or anti-correlated with the crystallization propensity and not with each other. These features were selected empirically using the Training data set. Three of these features are based on amino acid compositions, another three are based on free energy terms and two on hydrophobicity-based indices. The remaining three correspond to the instability index, distance of the first amino acid of medium polarizability from the N-terminus, and fraction of long segments (15AAs or longer) that are characterized by high amino acid complexity.

Supporting Figure S5 presents the box plots of values of the 11 features on the Training dataset along with their biserial correlation (with the binary crystallization output).

Hydrophobicity-based features (features which are based on MANP780101 "Average surrounding hydrophobicity" (Manavalan & Ponnuswamy, 1978) and CASG920101 "Hydrophobicity scale from native protein structures" (Casari & Sippl, 1992) indices) show that non-crystallizable proteins tend to have longer segments characterized by a wider range of hydrophobicity (lower values for the minimum in the MANP780101-based feature and higher values for the maximum in the CASG920101-based feature), whereas crystallizable proteins tend to exclude long segments with either high or low hydrophobicity. The hydrophobicity of the protein chain has been linked with crystallization outcome in many studies (Goh *et al*., 2004; Overton &Barton, 2006; Chen *et al*., 2007; Overton *et al*., 2008; Kurgan *et al*., 2009; Price *et al*., 2009; Babnigg & Joachimiak, 2010; Overton *et al*., 2011), and two of these studies also investigated hydrophobicity in segments of a protein sequence (Babnigg & Joachimiak, 2010; Mizianty & Kurgan, 2011).

The distribution of values of the three free energy-based features (features which are based on WERD780102 "Free energy change of epsilon(i) to epsilon(ex)" (Wertz & Scheraga, 1978), RADA880103 "Transfer free energy from vap to chx" (Radzicka & Wolfended, 1988), and WERD780103 "Free energy change of alpha(Ri) to alpha(Rh)" (Wertz & Scheraga, 1978) indices)

show that the non-crystallizable proteins are more likely to include segments with higher and lower free energy change values, whereas crystallizable proteins consist of regions with medium free energy change values; this is similar to the observation related to hydrophobicity. The indices related to the free energy changes were also used to design PPCpred (Mizianty & Kurgan, 2011) and MCSG Z-score (Babnigg & Joachimiak, 2010).

Crystallizable proteins are shown to be enriched in Glu, whereas high content of Ser and Cys is anti-correlated and is characteristic to proteins which are hard to crystallize. This agrees with observations in ref. (Babnigg & Joachimiak, 2010; Mizianty & Kurgan, 2011); the Glu content has been also used in ref. (Price *et al.*, 2009), whereas Ser and Cys contents were used in ref. (Overton *et al.*, 2008) and ref. (Overton *et al.*, 2008; Slabinski *et al.*, 2007), respectively.

Instability index, with higher values denoting instable proteins with shorter in vivo half-life, tends to be higher for the non-crystallizable proteins, which agrees with the finding in ref. (Slabinski *et al.*, 2007).

Crystallizable proteins are shown to have a large fraction of long high complexity segments predicted by SEG (Wootton & Federhen, 1993). In fact, over 50% of crystallizable proteins have no low complexity segments at all. Low complexity regions were linked to disorder, with a general rule that inclusion of a larger number and longer low complexity regions implies higher content of disorder (Romero *et al.*, 2001). Information about the predicted disorder was used to determine protein crystallizability in four previous studies (Slabinski *et al.*, 2007; Price *et al.*, 2009; Mizianty & Kurgan, 2011; Mizianty & Kurgan, 2012).

Interestingly, it seems that the non-crystallizable proteins have amino acids with medium polarizability (Cys, Pro, Asn, Val, Glu, Gln, Ile, Leu) closer to the N-terminus than the crystallizable targets. We hypothesize that this could be due to disorder of the protein's N-terminus or the influence of affinity tags or other N-terminal modifications.

Except for the last feature, the characteristics associated with the features utilized by fDETECT are well grounded in the literature and have been shown to be markers of crystallization outcomes. This study formulates a novel combination of these characteristics that can be calculated quickly and which offers competitive levels of predictive performance for the prediction of the crystallization propensity.

## Supporting references

Babnigg G, Joachimiak A. (2010). Predicting protein crystallization propensity from protein sequence. *J Struct Funct Genomics* **11**, 71–80.

Casari G, Sippl MJ. (1992). Structure-derived hydrophobic potential. Hydrophobic potential derived from X-ray structures of globular proteins is able to identify native folds. *J Mol Biol* **224**: 725–732.

Chen K, Kurgan L, Rahbari M. (2007). Prediction of protein crystallization using collocation of amino acid pairs. *Biochem Biophys Res Commun* **355**, 764–769.

Dosztányi Z. et al. (2005). IUPred: web server for the prediction of intrinsically unstructured regions of proteins based on estimated energy content. *Bioinformatics* **21**, 3433–3434.

Dubchak I. et al. (1999). Recognition of a protein fold in the context of the Structural Classification of Proteins (SCOP) classification. *Proteins* **35**, 401–407.

Goh C-S. et al. (2004). Mining the structural genomics pipeline: identification of protein properties that affect high-throughput experimental analysis. *J Mol Biol* **336**, 115–130.

Guruprasad K. et al. (1990). Correlation between stability of a protein and its dipeptide composition: a novel approach for predicting in vivo stability of a protein from its primary sequence. *Protein Eng* **4**, 155–161.

Ikai A. (1980). Thermostability and aliphatic index of globular proteins. *J Biochem* **88**, 1895–1898.

Kawashima S. et al. (2008). AAindex: amino acid index database, progress report 2008. *Nucleic Acids Res* 36, D202–D205.

Kurgan L. et al. (2009). CRYSTALP2: sequence-based protein crystallization propensity prediction. *BMC Struct Biol* 9, 50.

Manavalan P, Ponnuswamy PK. (1978). Hydrophobic character of amino acid residues in globular proteins. *Nature* **275**, 673–674.

Mizianty MJ, Kurgan L. (2011). Sequence-based prediction of protein crystallization, purification and production propensity. *Bioinformatics* **27**, i24–i33.

Mizianty MJ, Kurgan L. (2012). CRYSpred: accurate sequence-based protein crystallization propensity prediction using sequence-derived structural characteristics. *Protein Pept Lett* **19**, 40–49.

Overton IM, Barton GJ. (2006). A normalised scale for structural genomics target ranking: the OB-Score. *FEBS Lett* **580**, 4005–4009.

Overton IM, Padovani G, Girolami M, Barton GJ. (2008). ParCrys: a Parzen window density estimation approach to protein crystallization propensity prediction. *Bioinformatics* **24**: 901–907.

Overton IM, van Niekerk CAJ, Barton GJ. (2011). XANNpred: neural nets that predict the propensity of a protein to yield diffraction-quality crystals. *Proteins* **79**, 1027–1033.

Price WN et al. (2009). Understanding the physical properties that control protein crystallization by analysis of large-scale experimental data. *Nat Biotechnol* **27**, 51–57

Radzicka A, Wolfenden R. (1988). Comparing the polarities of the amino acids: side-chain distribution coefficients between the vapor phase, cyclohexane, 1-octanol, and neutral aqueous solution. *Biochemistry* **27**, 1664 – 1670.

Romero P. et al. (2001). Sequence complexity of disordered protein. *Proteins* **42**, 38–48.

Slabinski L et al. (2007). The challenge of protein structure determination - lessons from structural genomics. *Protein Sci* **16**, 2472–2482.

Tomii K, Kanehisa M. (1996). Analysis of amino acid indices and mutation matrices for sequence comparison and structure prediction of proteins. *Protein Eng* **9**, 27–36.

Wertz DH, Scheraga HA. (1978). Influence of water on protein structure. An analysis of the preferences of amino acid residues for the inside or outside and for specific conformations in a protein molecule. *Macromolecules* **11**, 9–15.

Wootton JC, Federhen S. (1993). Statistics of local complexity in amino acid sequences and sequence databases. *Comps Chem* **17**, 149–163.

**Table S1** Summary of the datasets used to design and evaluate fDETECT and to perform analysis of coverage by the X-ray structures.

| Dataset | # of proteins | Notes |
|---|---|---|
| Training | 3,587 | Used to design fDETECT; 1,204 crystallizable and 2,383 non-crystallizable chains. |
| Test | 3,584 | Used to evaluate and compare fDETECT with existing crystallization propensity predictors; 1,204 crystallizable and 2,380 non-crystallizable chains. |
| PDB | 50,138 | Non-redundant PDB chains with resolution no higher than 3.5 Å that were deposited between Jan 1st 1993 and Dec 31st 2012. |
| UniProt | 9,586,243 | 1,953 complete proteomes (106 archaea, 1043 bacteria, 265 eukaryotes and 539 viruses) from release 2012_07 of UniProt; 8,652,940 non-redundant proteins. |

**Table S2** Distribution of GO annotations, complete proteomes, protein sequences, non-redundant (nr) sequences, and modeling families (MFs).

We consider GO annotations that cover at least 20 MFs.

| | | All | Bacteria | Archaea | Eukaryota | Viruses | | | |
|---|---|---|---|---|---|---|---|---|---|
| | | | | | | All | Archaeal | Bacterial | Eukaryotic |
| All proteomes | # of proteomes | 1,953 | 1,043 | 106 | 265 | 539 | 8 | 54 | 477 |
| | # of sequences | 9,586,243 | 5,077,609 | 276,733 | 4,208,817 | 23,084 | 410 | 4,126 | 18,548 |
| | # of nr sequences | 8,652,940 | 4,284,068 | 260,871 | 4,087,314 | 21,093 | 410 | 3,734 | 16,949 |
| | # of MF | 1,734,048 | 896,393 | 85,145 | 867,451 | 11,492 | 402 | 2,850 | 8,261 |
| Proteomes with ≥ 100 MFs | # of proteomes | 1,486 | 1,041 | 106 | 265 | 37 | 0 | 8 | 29 |
| | # of sequences | 9,573,361 | 5,077,221 | 276,733 | 4,208,817 | 10,590 | 0 | 2,018 | 8,572 |
| | # of nr sequences | 8,641,375 | 4,283,680 | 260,871 | 4,087,314 | 9,778 | 0 | 1,802 | 7,976 |
| | # of MF | 1,730,635 | 896,300 | 85,145 | 867,451 | 6,757 | 0 | 1,621 | 5,145 |
| GO annotations with ≥ 20 MFs | # of annotations | 4,719 | 2,026 | 554 | 3,767 | 83 | | | |
| | # of sequences | 5,596,098 | 3,298,189 | 158,613 | 2,131,015 | 8,281 | | | |
| | # of nr sequences | 4,960,913 | 2,746,029 | 149,472 | 2,057,855 | 7,766 | | | |
| | # of MF | 622,279 | 348,743 | 34,305 | 268,989 | 3,209 | | | |

**Table S3** Evaluation of the considered designs based on five-fold cross validation of the Training dataset.

Results are sorted according to the AUC scores (the best value for each measure is given in bold) and the selected design is highlighted in grey.

| Method | Time per protein [s] | # of feat. | Accuracy | std | MCC | std | Specificity | std | Sensitivity | std | AUC | std |
|---|---|---|---|---|---|---|---|---|---|---|---|---|
| LIBSVM_RBF | 0.217 | 11 | 72.7% | 0.5% | 0.339 | .009 | 88.8% | 0.9% | 40.5% | 1.1% | **0.772** | .004 |
| **LOGISTIC** | 0.002 | 11 | 72.5% | 0.6% | 0.327 | .009 | 90.8% | 0.7% | 35.9% | 0.8% | 0.771 | .004 |
| LIBSVM_LIN | 0.163 | 11 | 72.4% | 0.6% | 0.334 | .007 | 87.5% | 1.0% | 41.9% | 1.3% | 0.769 | .004 |
| RBF NETWORK | 0.003 | 11 | **72.9%** | 0.7% | **0.359** | .011 | 85.1% | 1.1% | 48.3% | 1.3% | 0.769 | .006 |
| RBF NETWORK$^{fs}$ | 0.002 | 8 | 72.8% | 0.5% | 0.349 | .009 | 86.9% | 0.9% | 44.4% | 1.0% | 0.762 | .006 |
| LOGISTIC$^{fs}$ | **0.001** | 7 | 72.1% | 0.6% | 0.309 | .008 | **92.1%** | 0.7% | 31.7% | 1.3% | 0.756 | .005 |
| LIBSVM_POLY | 0.143 | 11 | 70.9% | 1.0% | 0.311 | .035 | 85.7% | 7.1% | 41.6% | 13.7% | 0.755 | .008 |
| LIBSVM_LIN$^{fs}$ | 0.099 | 6 | 71.6% | 0.4% | 0.303 | .010 | 89.3% | 0.8% | 35.9% | 1.7% | 0.753 | .004 |
| LIBSVM_SIG | 0.251 | 11 | 71.1% | 1.2% | 0.331 | .031 | 82.9% | 7.0% | 47.6% | 12.6% | 0.753 | .009 |
| LIBSVM_RBF$^{fs}$ | 0.186 | 8 | 69.9% | 1.1% | 0.313 | .040 | 80.2% | 8.9% | 49.3% | 15.7% | 0.746 | .012 |
| LIBSVM_POLY$^{fs}$ | 0.110 | 8 | 67.6% | 3.8% | 0.303 | .041 | 75.0% | 15.0% | **53.2%** | 21.2% | 0.743 | .014 |
| LIBSVM_SIG$^{fs}$ | 0.206 | 6 | 67.0% | 4.5% | 0.287 | .041 | 75.6% | 16.0% | 50.1% | 20.7% | 0.732 | .008 |

fs – with the wrapper-based feature selection

**Table S4** Parameters of the distributions of median crystallization propensity scores.

| | Archaea | Bacteria | Eukaryota |
|---|---|---|---|
| Mean | 0.394 | 0.329 | 0.138 |
| Standard deviation | 0.054 | 0.049 | 0.037 |
| Skewness | 0.171 | 0.269 | 1.340 |

**Table S5** List of G-Protein Coupled Receptors (GPCRs) with the highest predicted crystallization propensities.

| UniPRotID | Protein name | Organism | Score |
|---|---|---|---|
| B0WAX1 | Olfactory receptor | Culexquinquefasciatus (Southern house mosquito) | 0.435 |
| D6WG06 | Gustatory receptor 30 | Triboliumcastaneum (Red flour beetle) | 0.415 |
| Q7PK67 | AGAP009706-PA | Anopheles gambiae (African malaria mosquito) | 0.385 |
| B4QNG4 | GD12418 | Drosophila simulans (Fruit fly) | 0.366 |
| Q9VVF3 | Putative odorant receptor 74a | Drosophila melanogaster (Fruit fly) | 0.365 |
| D6WC28 | Gustatory receptor 203 | Triboliumcastaneum (Red flour beetle) | 0.344 |
| D6WXW3 | Gustatory receptor 191 | Triboliumcastaneum (Red flour beetle) | 0.341 |
| B0XLS6 | Odorant receptor 83c | Culexquinquefasciatus (Southern house mosquito) | 0.335 |
| H0XVV2 | Uncharacterized protein | Otolemurgarnettii (Small-eared galago) | 0.333 |
| H0Y166 | Uncharacterized protein | Otolemurgarnettii (Small-eared galago) | 0.329 |
| B4H6K7 | GL15497 | Drosophila persimilis (Fruit fly) | 0.325 |
| Q2LZG6 | Or74a | Drosophila pseudoobscurapseudoobscura (Fruit fly) | 0.324 |
| G3IPA0 | Vomeronasal type-2 receptor 26 | Cricetulusgriseus (Chinese hamster) | 0.322 |
| G3U577 | Uncharacterized protein | Loxodontaafricana (African elephant) | 0.319 |
| G1U260 | Uncharacterized protein | Oryctolaguscuniculus (Rabbit) | 0.315 |
| Q7PSF0 | AGAP009394-PA | Anopheles gambiae (African malaria mosquito) | 0.312 |
| B3NLE6 | GG21080 | Drosophila erecta (Fruit fly) | 0.307 |
| F7CRM9 | Uncharacterized protein | Ornithorhynchusanatinus (Duckbill platypus) | 0.306 |
| Q29H44 | Or9a | Drosophila pseudoobscurapseudoobscura (Fruit fly) | 0.305 |
| B0XGA0 | Odorant receptor 83c | Culexquinquefasciatus (Southern house mosquito) | 0.305 |
| B0VXA6 | Olfactory receptor 599 | Callithrixjacchus (White-tufted-ear marmoset) | 0.305 |
| D6W8K5 | Gustatory receptor 165 | Triboliumcastaneum (Red flour beetle) | 0.303 |
| B4N5C3 | GK20347 | Drosophila willistoni (Fruit fly) | 0.302 |
| Q17NP3 | AAEL000614-PA | Aedesaegypti (Yellowfever mosquito) | 0.302 |
| B4GD96 | GL11721 | Drosophila persimilis (Fruit fly) | 0.301 |

**Table S6** Division of AAs into groups based on their physicochemical and structural properties.

| Characteristic | AA groups | | |
|---|---|---|---|
| Hydrophobicity | Polar<br>R, K, E, D, Q, N | Neutral<br>G, A, S, T, P, H, Y | Hydrophobicity<br>C, L, V, I, M, F, W |
| Normalized van der Waals volume | [0 – 2.78]<br>G, A, S, T, P, D | [2.95-4.0]<br>N, V, E, Q, I, L | [4.03 – 8.08]<br>M, H, K, F, R, Y, W |
| Polarity | [4.9 – 6.2]<br>L, I, F, W, C, M, V, Y | [8.0 – 9.2]<br>P, A, T, G, S | [10.4 – 13.0]<br>H, Q, R, K, N, E, D |
| Polarizability | [0 – 1.08]<br>G, A, S, D, T | [0.128 – 0.186]<br>C, P, N, V, E, Q, I, L | [0.219 – 0.409]<br>K, M, H, F, R, Y, W |
| Charge | Positive<br>K, R | Neutral<br>A, N, C, Q, G, H, I, L, M, F, P, S, T, W, Y, V | Negative<br>D, E |
| Secondary structure | Helix<br>E, A, L, M, Q, K, R, H | Strand<br>V, I, Y, C, W, F, T | Coil<br>G, N, P, S, D |
| Solvent accessibility | Buried<br>A, L, F, C, G, I, V, W | Exposed<br>R, K, Q, E, N, D | Intermediate<br>M, P, S, T, H, Y |

**Table S7** List of considered 64 hydrophobicity- and energy-based indices.

The names are based to the nomenclature from the AAIndex1 database.

| | | | | |
|---|---|---|---|---|
| ARGP820101 | BULH740101 | CHAM820102 | CIDH920105 | EISD840101 |
| EISD860101 | EISD860102 | EISD860103 | FAUJ830101 | GOLD730101 |
| GUYH850101 | HOPT810101 | JANJ790102 | JOND750101 | KYTJ820101 |
| LAWE840101 | LEVM760101 | MANP780101 | MIYS850101 | NOZY710101 |
| OOBM770101 | OOBM770102 | OOBM770103 | OOBM770104 | OOBM770105 |
| OOBM850103 | OOBM850104 | PONP800101 | PONP800102 | PONP800103 |
| PRAM900101 | RADA880101 | RADA880102 | RADA880103 | RADA880104 |
| RADA880105 | ROBB790101 | ROSM880101 | ROSM880102 | SIMZ760101 |
| SWER830101 | VHEG790101 | WERD780102 | WERD780103 | WERD780104 |
| YUTK870101 | YUTK870102 | YUTK870103 | YUTK870104 | ZIMJ680101 |
| PONP930101 | WILM950101 | WILM950102 | WILM950103 | WILM950104 |
| KUHL950101 | JURD980101 | WOLR790101 | KIDA850101 | COWR900101 |
| BLAS910101 | CASG920101 | ENGD860101 | FASG890101 | |

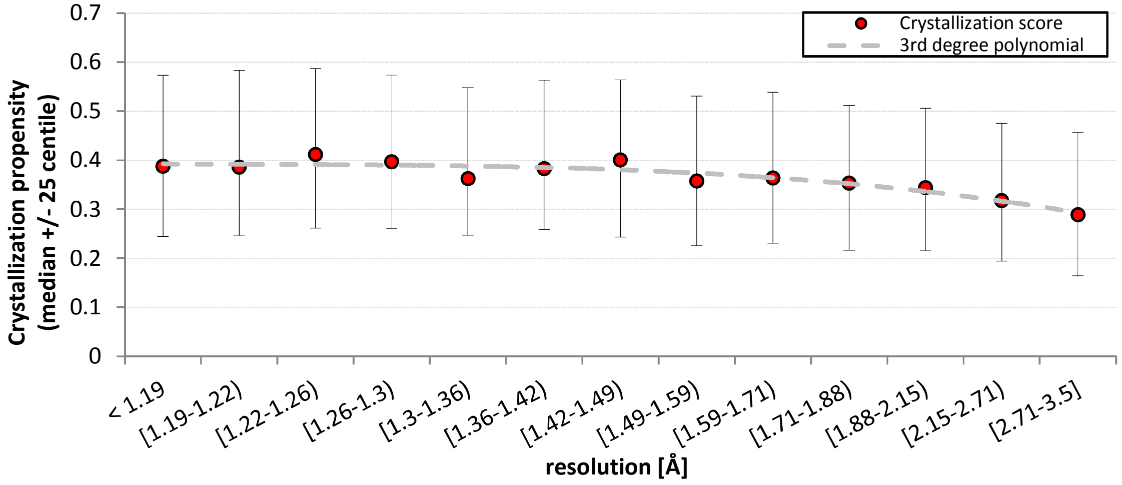


**Figure S1** Relation between predicted crystallization propensity and crystal resolution. The box plot shows 25th, 50th, and 75th percentile of scores for the crystal structures with resolution from a given range. Ranges for resolutions were selected to reflect the inverted cubical nature of crystal diffraction resolution. Dashed line represents fitted 3rd degree polynomial.

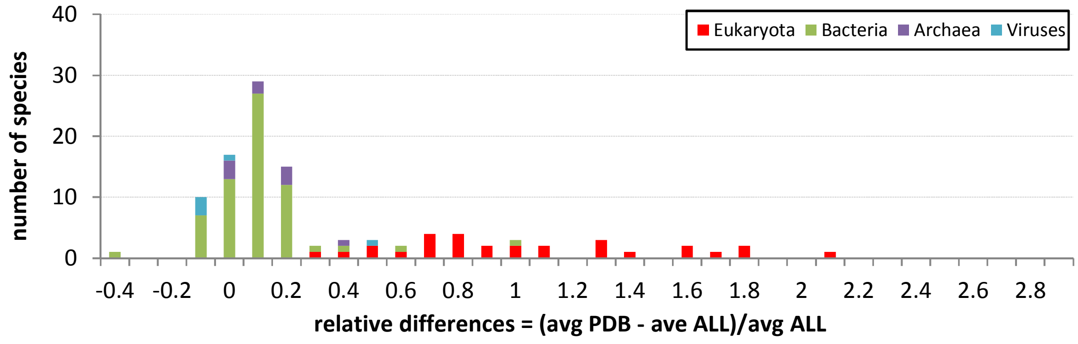


**Figure S2** The relative difference in crystallization score between the PDB and UniProt. Graph compares the predicted crystallization propensity for all proteins from a given proteome and the proteins from a given proteome which were deposited in the PDB. We selected proteomes with at least 20 chains deposited in the PDB.

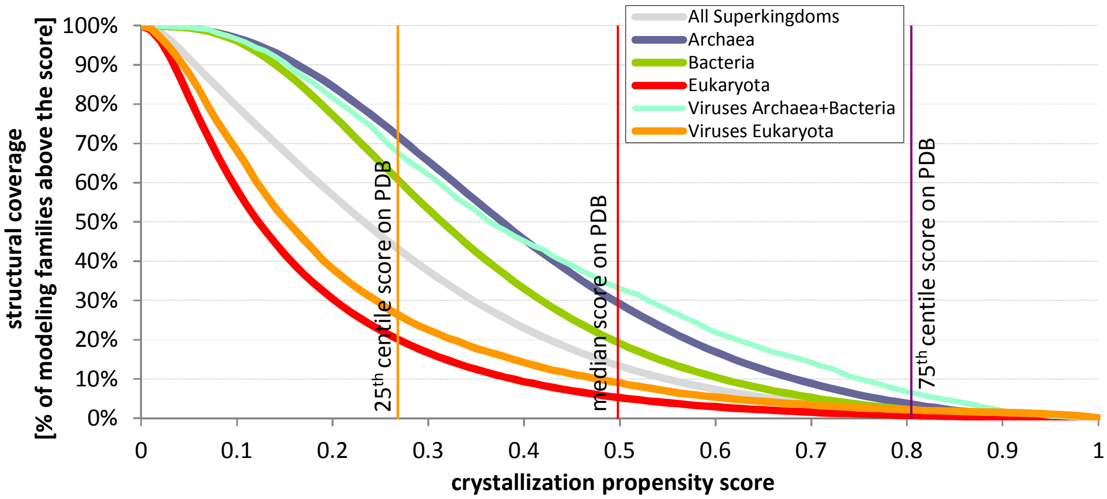


**Figure S3** Coverage that is attainable by X-ray crystallography for proteins in all considered complete proteomes, in eukaryotes, bacteria, archaea, and viruses. The vertical lines show the cut-off values that correspond to 25th centile, median and 75th centile of the crystallization propensity scores of proteins from the PDB dataset.

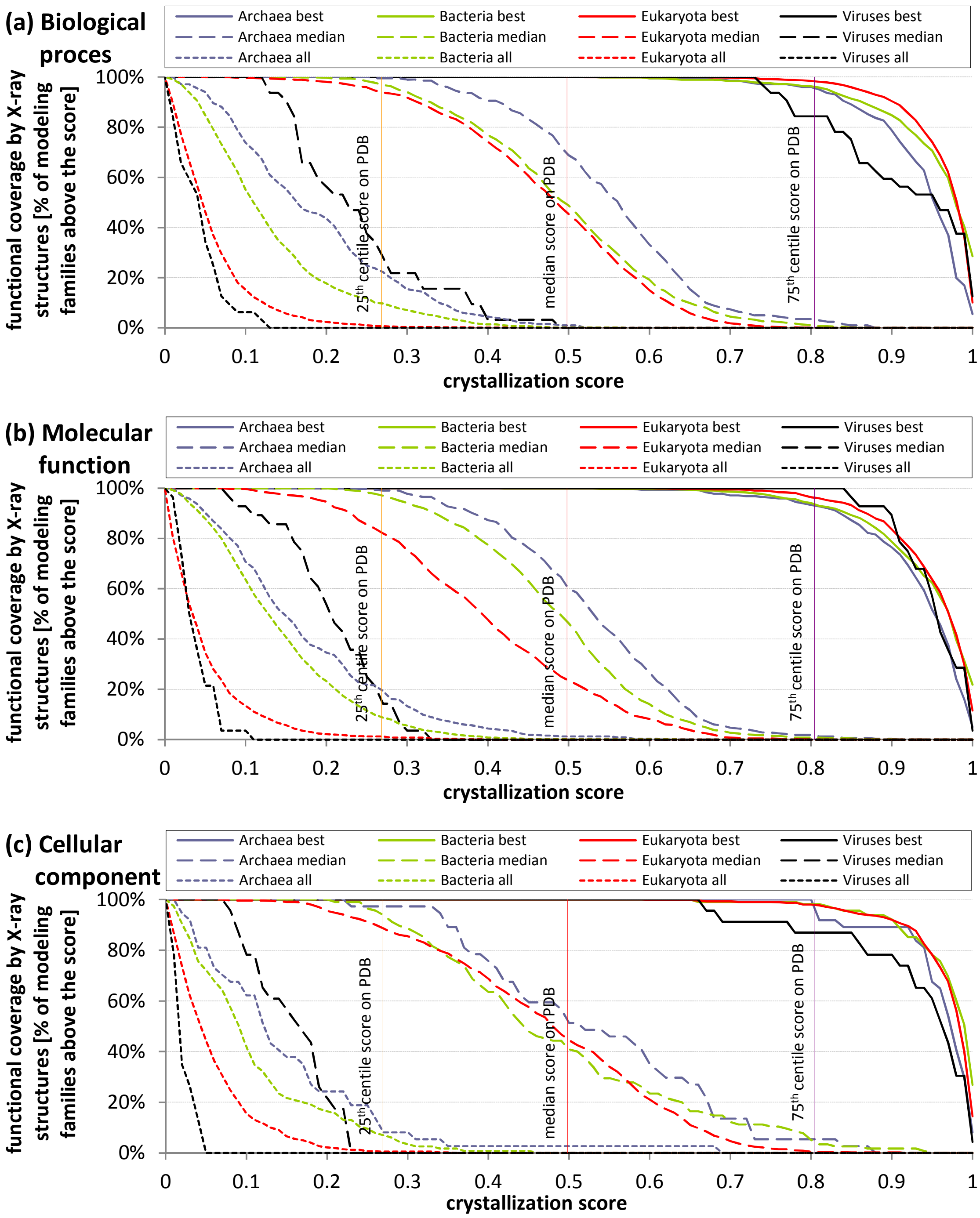


**Figure S4** The functional coverage (number of GO annotations with X-ray structures divided by the number of all available GO annotations in a given superkingdom) of the considered 4,719 GO annotations grouped into the three superkingdoms of life and viruses. (a), (b), and (c) show results for the biological processes, molecular functions and cellular components types of annotations, respectively. Proteins with a given GO annotations were mapped into modelling families. A given modelling family can be structurally covered if it includes at least one protein with a crystallization propensity above a cut-off value provided on the x-axis; the remaining structures in that family can be

obtained using homology modelling. The solid lines assume that a given GO annotation is covered when one or more of its annotated modelling families has an attainable structure. The dashed/dotted lines assume that a given annotation is covered when at least 50%/all of its modelling families are structurally covered. The vertical lines show the cut-off values that correspond to 25$^{th}$ centile, median and 75$^{th}$ centile of the crystallization propensity scores of the clustered proteins from the PDB dataset. To assure statistically sound estimates and to accommodate for the incompleteness of the GO annotations, we limited analysis to the annotations with at least 20 modelling families.

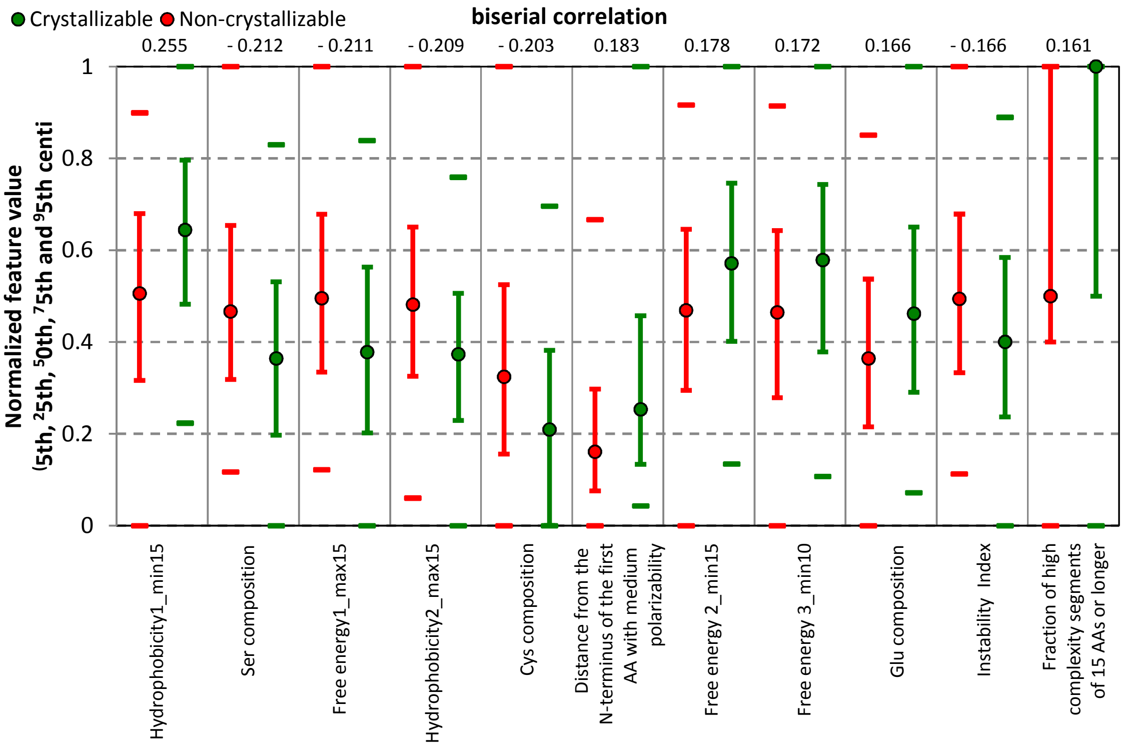


**Figure S5** Distribution of normalized features' values on the Training dataset. Values were normalized using min-max normalization where 5$^{th}$ and 95$^{th}$ centile values were selected as minimal and maximal values, respectively. The box plots show the median (50$^{th}$ centile, hollow circle), 25 and 75$^{th}$ centiles (whiskers) and the min and max values (dash markers). Features are sorted from left to right according to their absolute biserial correlation, shown at the top of the figure in the descending order. Hydrophobicity1 refers to MANP780101 index, Hydrophobicity2 to CASG920101 index, Free energy1 to WERD780102 index, Free energy2 to RADA880103 index, and Free Energy3 to WERD780103 index. Indices were taken from the AAIndex database. minX and maxX refer to minimal and maximal average value of a given index over possible segments of X neighboring residues in the sequence.
